# Structure vs. chemistry: Alternate mechanisms for controlling leaf microbiomes

**DOI:** 10.1371/journal.pone.0275734

**Published:** 2023-03-21

**Authors:** Kenny J. X. Lau, Elena S. Gusareva, Irvan Luhung, Balakrishnan N. V. Premkrishnan, Anthony Wong, Tuang Yeow Poh, Akira Uchida, Elaine L. Oliveira, Daniela I. Drautz-Moses, Ana Carolina M. Junqueira, Stephan C. Schuster

**Affiliations:** 1 Singapore Centre for Environmental Life Sciences Engineering, Nanyang Technological University, Singapore, Singapore; 2 The Asian School of the Environment, Nanyang Technological University, Singapore, Singapore; 3 Translational Respiratory Research Laboratory, Lee Kong Chian School of Medicine, Nanyang Technological University, Singapore, Singapore; 4 Departamento de Genética, Instituto de Biologia, Universidade Federal do Rio de Janeiro, Rio de Janeiro, Brazil; City University of New York, UNITED STATES

## Abstract

The analysis of phyllosphere microbiomes traditionally relied on DNA extracted from whole leaves. To investigate the microbial communities on the adaxial (upper) and abaxial (lower) leaf surfaces, swabs were collected from both surfaces of two garden plants, *Rhapis excelsa* and *Cordyline fruticosa*. Samples were collected at noon and midnight and at five different locations to investigate if the phyllosphere microbial communities change with time and location. The abaxial surface of *Rhapis excelsa* and *Cordyline fruticosa* had fewer bacteria in contrast to its adaxial counterpart. This observation was consistent between noon and midnight and across five different locations. Our co-occurrence network analysis further showed that bacteria were found almost exclusively on the adaxial surface while only a small group of leaf blotch fungi thrived on the abaxial surface. There are higher densities of stomata on the abaxial surface and these openings are vulnerable ports of entry into the plant host. While one might argue about the settling of dust particles and microorganisms on the adaxial surface, we detected differences in reactive chemical activities and microstructures between the adaxial and abaxial surfaces. Our results further suggest that both plant species deploy different defence strategies to deter invading pathogens on the abaxial surface. We hypothesize that chemical and mechanical defence strategies evolved independently for harnessing and controlling phyllosphere microbiomes. Our findings have also advanced our understanding that the abaxial leaf surface is distinct from the adaxial surface and that the reduced microbial diversity is likely a consequence of plant-microbe interactions.

## Introduction

Leaves constitute a substantial surface area of the phyllosphere. The total area of combined adaxial and abaxial leaf surfaces is estimated to be 1,017,260,200 km^2^ globally [1]. Leaf surface is a habitat for bacteria and fungi from the phyla Bacteroidetes, Actinobacteria, Ascomycota and Basidiomycota [1–4]. Leaf-associated microbiomes may play an essential role in protecting plants against invasive phytopathogens [5]. Plants have also evolved dynamic physical barriers [6] and innate immune defence[7] mechanisms to avoid colonisation by phytopathogens. Leaf adaxial and abaxial surfaces have distinct anatomical structure and function. The adaxial surface is adapted for efficient light capture, while the abaxial surface is adapted for gaseous exchange during respiration [8]. In addition, the adaxial surface usually has a layer of cuticular wax to prevent water loss [9]. Some plants also have an epidermal outgrowth of trichomes on their leaves as a form of mechanical defence [10]. The abaxial leaf surface usually has a higher density of stomata and it has been well-established that as part of the plant’s innate immunity, the guard cells surrounding the stomatal pore will close upon detection of microbes [11].

Previous studies on the phyllosphere microbiome have employed a processing pipeline consisting of bulk whole-leaf sampling, washing, sonication and filtering, followed by DNA extraction and amplicon sequencing [12–14]. This process, however, aggregates the total microbial communities found on both adaxial and abaxial leaf surfaces. Furthermore, the amplicon sequencing technique might give rise to amplification biases [15, 16]. Therefore, metagenomic shotgun sequencing has been preferentially used in our study, to enable the simultaneous detection of both bacteria and fungi without the need for separate 16S, 18S and internal transcribed spacer (ITS) primers. Thus, unlike amplicon sequencing, the relative abundances of microbes were measured on the same scale. To segregate microbial communities from the adaxial and abaxial leaf surfaces, we conducted whole genome shotgun metagenomics analysis of swabs made on both surfaces of the two plant species, *Rhapis excelsa* and *Cordyline fruticosa*. These plants have clusters of broad leaves accessible for swabbing. *R*. *excelsa* is native of southern China and Southeast Asia [17], and belongs to the *Arecaeae* family, which also consists of agricultural plant species such as coconut (*Cocosnucifera spp*.), date palm (*Phoenix dactylifera*) and oil palm (*Elaeis guineensis*). *C*. *fruticosa*, on the other hand, belongs to the *Agavaceae* family and is a native of tropical Asia, Australia and Pacific Islands, including Hawaii [18].

The microbial leaf communities were collected using swabs, which have widely been used in medical research and forensics, but only few plant studies collected swabs from leaves for microbiome analysis [19, 20]. The metagenomic dataset generated from the leaf swabs can be used to address overarching hypotheses involving site-specific and temporal diurnal effects on the relative composition of the phylloplane microbiome. In this study, we show that the adaxial and abaxial leaf surfaces harbour distinct microbial communities and our results further suggest that the two plants may have deployed different defence strategies to control the microbiomes on their leaf surfaces.

## Methods

### Leaf swabbing

Leaves of the species *R*. *excelsa* and *C*. *fruticosa* from different locations in Nanyang Technological University (NTU), Singapore, were selected for swabbing (S1 Table). To improve the DNA yield, each sample consisted of swabbing surfaces of ten leaves of the same plant. Sampling was done on two different days and times, 31 October 2017 between 11 pm and 1 am and 22 November 2017 between 11 am and 1pm. Samples were collected at midnight and noon to see if they have a diurnal cycle like the airborne microbial communities in tropical air [21]. Two plants of each species were sampled at each location and they were all outdoor plants. The adaxial and abaxial surfaces of *R*. *excelsa* and *C*. *fruticosa* leaves were swabbed with 4N6Floq swabs (Copan, USA) dipped in Gibco phosphate buffered saline (PBS), pH 7.2 (Life Technologies, USA) with 0.1% Triton-X100 (Sigma-Aldrich, USA). The complete experimental processing pipeline is illustrated in S1 Fig and the average number of reads outlined in S2 Table. The reagent blank samples were collected at the sampling site. We performed dipping a sterile swab in Phosphate Buffer Saline. The blanks were then treated the same as other swab samples collected.

### Taxonomical identification of plants

The taxonomical identification of the plant species from each location was confirmed using DNA barcoding and key anatomical structures [22, 23]. DNA from plant leaves were extracted using DNeasy PowerWater kit (Qiagen, Germany) according to manufacturer’s protocol and subsequently amplified with polymerase chain reaction (PCR) targeting the *rbcL* (RUBISCO large subunit) and *MatK* (Maturase K) genes. The *rbcL* primer sets used were *rbcLa-F* (5’- ATGTCACCACAAACAGAGACTAAAGC-3’) [24] and *rbcLa-R* (5’-GTAAAATCAAGT CCACCRCG-3’) [25], while the *MatK* primer sets used were *MatK-xF* (5’- TAATTTACGATCAATTCATTC-3’) [26] and *MatK-MALP* (5’-ACAAGAAAGTCGAAGTAT-3’) [27]. PCR conditions were as follows: 2× KAPA HiFi Hotstart Ready Mix (12.5 μl) (Kapa Biosystems, USA), primers (for both forward and reverse primers each 0.75 μl of 10 μM), template DNA (1 μl of 5 ng/μl) and H_2_O (10 μl) for each reaction (25 μl). PCR cycles for *rbcL* were performed as follows: 94°C for 3 min, 35 cycles of 94°C for 30 s, 52°C for 40 s, 60°C for 1 min with a final elongation step at 60°C for 5 min while PCR cycles for *MatK* were as follows: 98°C for 45 s, 35 cycles of 98°C for 10 s, 54°C for 30 s, 72°C for 40 s with a final elongation step at 72°C for 10 min. The PCR products were sent to AITbiotech Singapore for Sanger sequencing. Read quality was analysed with 4Peaks (Mekentosj, Amsterdam) and Chromas (Technelysium, Australia) where sites below Phred score 20 were filtered and trimmed. Forward and reverse reads were overlapped using BioEdit v7.2.6.1 [28]. Sequence similarity results correspond to *Rhapis* and *Cordyline* genus using blastn [29] and BOLD [30] at 99% identity and 100% query cover respectively. Morphological descriptions by Hastings [22] and Gilbert [23] helped to further validated the taxonomic identification of the plants as *Rhapis excelsa* and *Cordyline fruticosa*.

### DNA extraction, library preparation and sequencing

After sampling, the swabs were snapped into a DNeasy PowerWater kit (Qiagen, Germany) bead tube and immediately transported to the laboratory for DNA extraction following manufacturer’s instructions with the addition of 0.1 mg/ml proteinase K (Sigma-Aldrich, USA) and overnight sonication at 65°C [31]. Extracted DNA samples were quantitated on a Qubit 2.0 fluorometer, using the Qubit dsDNA High Sensitivity Assay Kit (Invitrogen, USA). The DNA was sheared and size-selected to an average insert size of 450 bp. More than 2 million reads per library were generated on a HiSeq 2500 sequencer (Illumina, USA). In detail, high-throughput sequencing libraries were prepared with the Accel-NGS 2S Plus DNA Kit (Swift Biosciences), following the manufacturer’s instructions provided with the kit. The starting amount of DNA for library preparation was normalized to 5 ng. DNA shearing was performed on a Covaris E220 focused-ultrasonicator. Library quantitation was performed using Promega’s QuantiFluor dsDNA assay and the average library size was determined by running the libraries on a Tapestation D1000 Screentape (Agilent). Library concentrations were normalized to 4nM and the concentration was validated by qPCR on a QuantStudio-3 real-time PCR system (Applied Biosystems), using Kapa Biosystem’s Library Quantification for Illumina platforms.

### Bioinformatics and taxonomic assignment

The FASTQ files obtained from sequencing were first examined for quality using FastQC (version 0.11.5) [32]. Raw sequence reads with minimum length of 250 bp with an error probability of less than 0.05 and a per base sequence quality score of at least 20 were selected. Adapter sequences were trimmed using Cutadapt (version 1.15) [33]. Two million reads per sample were randomly sub-sampled to represent the entire population of reads to speed up the computing process. This step was verified with a rarefaction curve to ensure that no new species were observed when sampling beyond the two million reads (S2 Fig). The reads were then queried across NCBI’s non-redundant (nr) protein database using RAPSearch2 [34]. All output files including the swab and reagent blank controls were exported from MetaGenome ANalyzer (MEGAN) v6.12.0 [35] in absolute read counts. Fungi and bacteria species-level taxonomy tables were imported as text files for subsequent removal of contaminating taxa that could have originated from the swab itself or the reagent blank using the *decontam* [36] package in R [37]. Microbial species that had less than 25 read counts were also removed. The decontaminated files were then re-imported into MEGAN and normalized to match the sample with the lowest total read count for comparative metagenomic analyses and visualisation. To study potential gene functions, reads were also mapped to the Kyoto Encyclopaedia of Genes and Genomes (KEGG) pathways in MEGAN and plotted as heatmap of normalised reads based on z-scores.

### Statistical analyses

The taxonomy tables were exported from MEGAN for subsequent statistical analysis in R using *vegan* [38] and *phyloseq* [39] packages. Beta-diversity was assessed using the Bray-Curtis dissimilarity metric. Principal coordinate analysis (PCoA) plots were created to observe clustering patterns in relation to spatiotemporal factors namely, the adaxial/abaxial surface, time of the day and locations. Analysis of similarity (ANOSIM) was performed using the anosim function in *vegan*. Alpha-diversity was analysed using both Shannon and Chao-1 diversity index. The Chao-1 index was plotted as scatter plots and Mann-Whitney test was performed in PRISM v7.03 [40].

In addition, co-occurrence network analysis of microbial species was performed with Spearman rank correlation cut-off of 0.8 and p-value of 0.01 adapted from a script written by Li, et al. [41] in R. The co-occurrence network was then visualised using Gephi v0.9.2 [42]. The relative abundance of the clusters of co-occurring microbes generated with Gephi was plotted with *ggplot2* [43] package in R. The list of all microbial species identified in both *R*. *excelsa*, *C*. *fruticosa* and in the co-occurrence analysis were extracted and plotted in Venn diagrams to examine the proportional similarity of clusters using *venndiagram* [44] package in R.

### Processing of leaf samples for scanning electron microscopy (SEM)

The leaves were fixed in 2.5% glutaraldehyde in phosphate buffer. Samples were washed gently with distilled water, placed onto an aluminium stub sample mount using double sided carbon tape and snap frozen in liquid nitrogen. After metal block cooling, equilibrated metal blocks were freeze-dried using custom-made freeze-drying apparatus. The metal block with samples was immediately transferred to Leica SCD050 (Leica, Germany) sputter coater stage lined with a layer of insulating foam and the chamber was promptly evacuated. Samples were slowly freeze dried in the evacuated chamber overnight, then removed and sputter coated with 4 nm layer of platinum. Scanning electron microscopy (SEM) analysis was then performed using a Jeol JSM-5690LV SEM.

### Reactive oxygen species (ROS) assay

Duplicate swab samples were made at each site. As a result, a collective of ten replicates were generated for *Rhapis excelsa* and another ten for *Cordyline fruticosa* across the different locations. The swab was collected using the same method as the metagenomic samples but Triton-X100 was not added to the swab buffer since surfactants may interfere with the cellular ROS activity [45]. The swab tips were resuspended in 500 μl of PBS in 1.5 ml amber tubes and samples were kept on ice and processed on the same day of sample collection. We modified an *in vivo* ROS assay protocol by B Nemzer, Z Pietrzkowski, T Chang and B Ou [46] for our leaf swabs. Briefly, samples were vortexed and centrifuged at 10,000 rpm at 4°C for 3 min. Subsequently, 240 μl of supernatant were transferred into a clean Eppendorf tube and 720 μl of methanol (Fisher Scientific, USA) was added to supernatant to precipitate proteins. The samples were vortexed briefly for 30 s and centrifuged at 10,000 rpm at 4°C for 5 min. Supernatants were transferred into clean tubes and the methanol was evaporated to dryness using Eppendorf Concentrator plus (Eppendorf, Germany). The evaporated samples were reconstituted in 60 μl PBS. A total of 40 μl of the reconstituted samples was loaded into each well of a black flat bottom 96 well plate (Nunc, Denmark) and 60 μl of dihydrorhodamine 123 dye (Invitrogen, USA) diluted with PBS to a working concentration of 202 μM with PBS were added to the samples. The fluorescence intensity was measured using Synergy H1 microplate reader (BioTek, USA) with an excitation wavelength of 485 nm and emission wavelength of 545 nm after 5 minutes. Bar charts were plotted in PRISM v7.03 [40] for visualisation.

### Surface water contact angle measurement

To measure the surface wettability of the leaves, the sessile drop method was performed at room temperature using the OCA-20 contact angle meter (DataPhysics, USA). The adaxial and abaxial leaves of both plants were cut into 25 mm^2^ squares and mounted onto microscope slides placed on the stage. A robotic arm dispensed 3 μl of a water droplet, which was suspended on the needle tip of the robotic syringe. The stage was then manually adjusted upwards until the specimen was in contact with the water droplet. The stage was then lowered slightly, and the water droplet-on-leaf image was captured. Using the instrument SCA software (DataPhysics, USA), a base line was drawn, and an ellipse was fitted over the water droplet for calculation of the contact angle.

## Results

### Metagenomic profiles of different leaf surfaces

**Fig 1:**
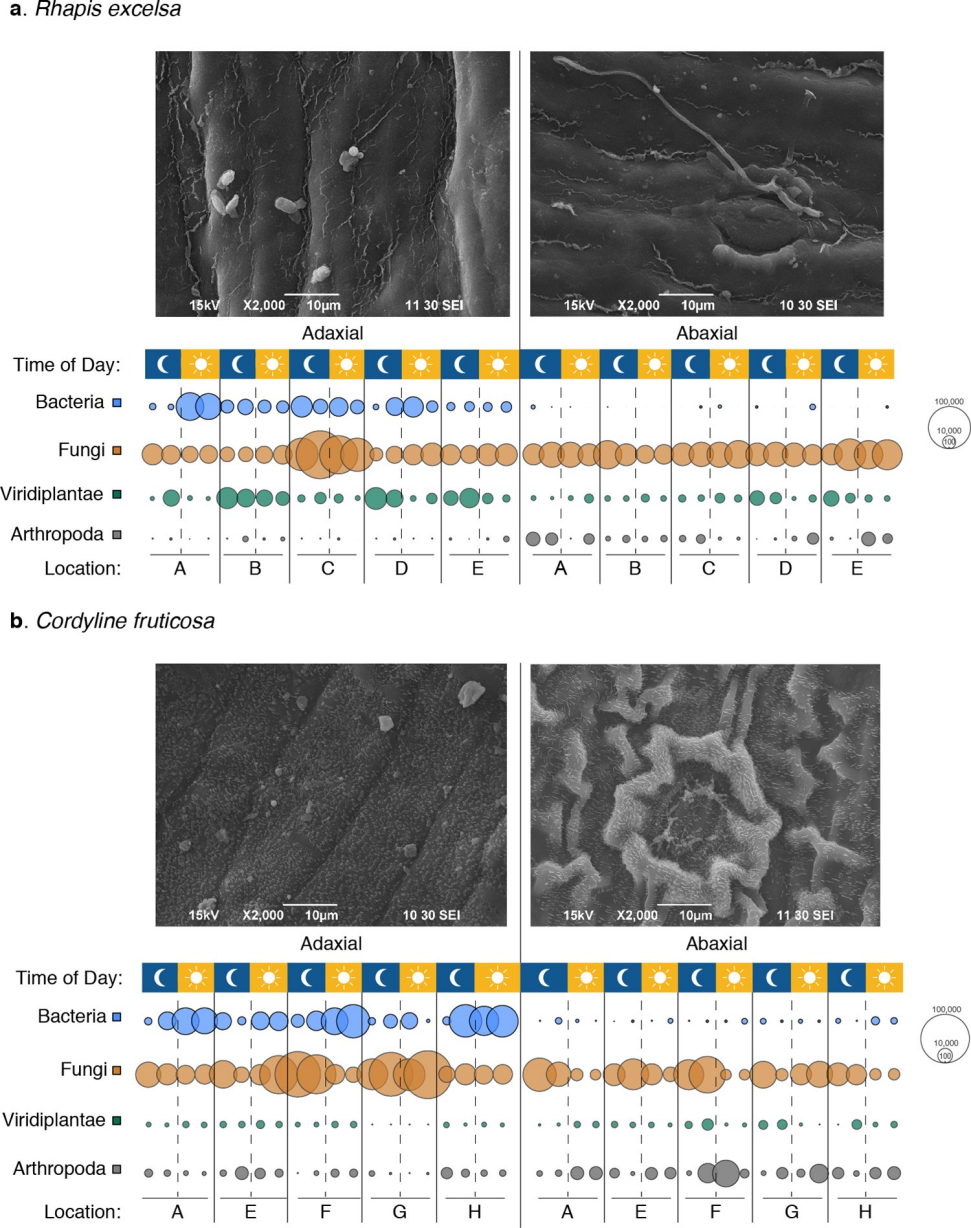
SEM images and bubble charts of microbes, plants and arthropod-related reads on the adaxial and abaxial leaf surfaces. The bubble sizes correspond to the relative abundance of reads assigned to bacteria, fungi, plant host and arthropods in the comparative metagenomic analyses. Reduction in bacterial read counts observed on the abaxial surface as compared to the adaxial surface in contrast to Fungi, Viridiplantae and Arthropoda reads on the leaf surfaces of (a) *R*. *excelsa* and (b) *C*. *fruticosa*. The moon and sun symbols refer to the time of sampling (midnight and noon), while the letters refer to the sampling location in S1 Fig. The SEM images were original images captured by Chee Peng Ng and David Liebl from the IMB-IMCB Joint Electron Microscopy Suite (A*STAR Singapore).

**Fig 2:**
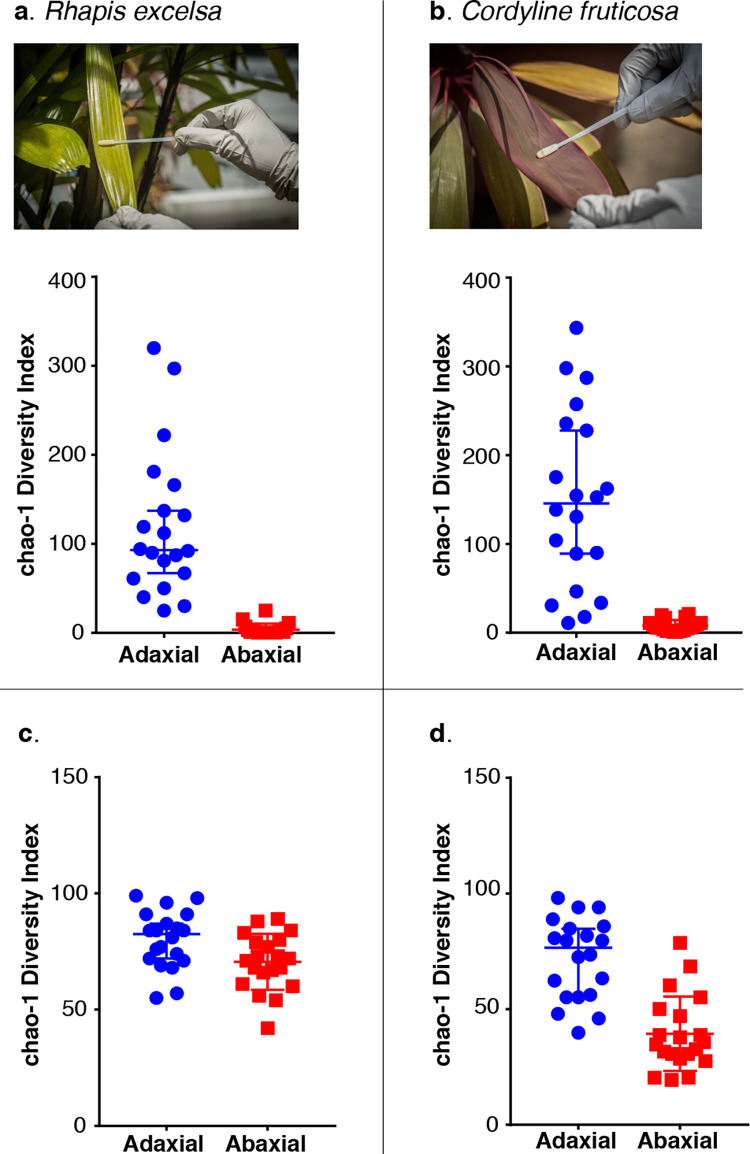
Dot plots of species richness using Chao-1 index of the adaxial and abaxial leaf surface. Approximately 95–98% fewer in bacterial species were observed on the abaxial leaf surface of (a) *R*. *excelsa* and (b) *C*. *fruticosa* in contrast to their adaxial surface. There was also a decrease in fungal species observed on the abaxial surface of (c) *R*. *excelsa* and (d) *C*. *fruticosa*. All four dot plots showed statistically significant differences between the adaxial and abaxial surface using Mann Whitney test (S2 Table). The photographs were taken by Balakrishnan N.V. Premkrishnan.

Our results consistently showed a decrease in microbial diversity, especially in the bacterial taxa, on the abaxial surface in both plants and across all locations. Our metagenomics analysis indicated a decrease in bacterial read counts on the abaxial surface as compared to their corresponding adaxial surface in both *R*. *excelsa* and *C*. *fruticosa*. This observation was consistent between two different times of the day (noon and midnight) as well as at five different locations (Fig 1A and 1B). The bacterial read counts ranged between 8,000 and 50,000 on the adaxial surface and between 100 and 3,000 on the abaxial surface. This observation was further supported by the dot plots of samples using the species richness estimate indicator, Chao-1 index (Fig 2). This reduction also held true for both bacterial and fungal species richness on the abaxial surface as compared to the adaxial surface when using the Mann Whitney test (S3 Table). The reduction in bacterial read counts was also eminent when we compared the top 40 bacterial species with the top 40 fungal species in both plants (S3 and S4 Figs).

Similarly, principal coordinate analysis (PCoA) plots using Bray-Curtis dissimilarity metric revealed two distinct clusters, corresponding to the adaxial and abaxial leaf surfaces (S5A Fig). Analysis of similarities (ANOSIM) statistical test showed that the two clusters were significantly different with a *p*-value of 0.001 and an *R*-statistic of 0.7322 for *R*. *excelsa* leaves and *R-*statistic of 0.717 for *C*. *fruticosa*. The segregation of the two clusters along PCoA1 suggests that the adaxial and abaxial surface accounts for the main difference observed and this may be driven mainly by the significant reduction in bacterial read counts on the abaxial surface.

The same PCoA analysis did not show any clustering of the data by the factors of time (sampling during day/night) or plants location (sampling site) (S6B and S6C Fig). Thus, sampling time and location have no influence on the leaf microbiome. However, when the samples were clustered according to plant species, distinct clustering between *Rhapis* and *Cordyline* plants (S6D Fig) was observed which shows that the two plant leaves have slightly different microbial communities. This difference is highlighted among *Aureobasidium spp*. where *Cordyline* has at least ten-fold higher than *Rhapis* and bacterial species in cluster B on the adaxial surface of *Cordyline* plant, which will be discussed in the next section of the co-occurrence analysis.

Lastly, we probe for functional genes by mapping the reads to the KEGG database in MEGAN to shed light on possible cellular activities that may be associated with the leaf microbiome. The heatmap in S7 Fig reveals that the adaxial leaf surfaces on both *Rhapis* and *Cordyline* plants have more reads that are related to cell growth, motility, membrane transport, catabolism, metabolism, and cell death with higher z-scores as compared to the abaxial surface. This observation suggests that there may be an active microbial community present on the adaxial surface that is not seen on the abaxial side.

### Co-occurrence analysis of leaf microbial communities

**Fig 3:**
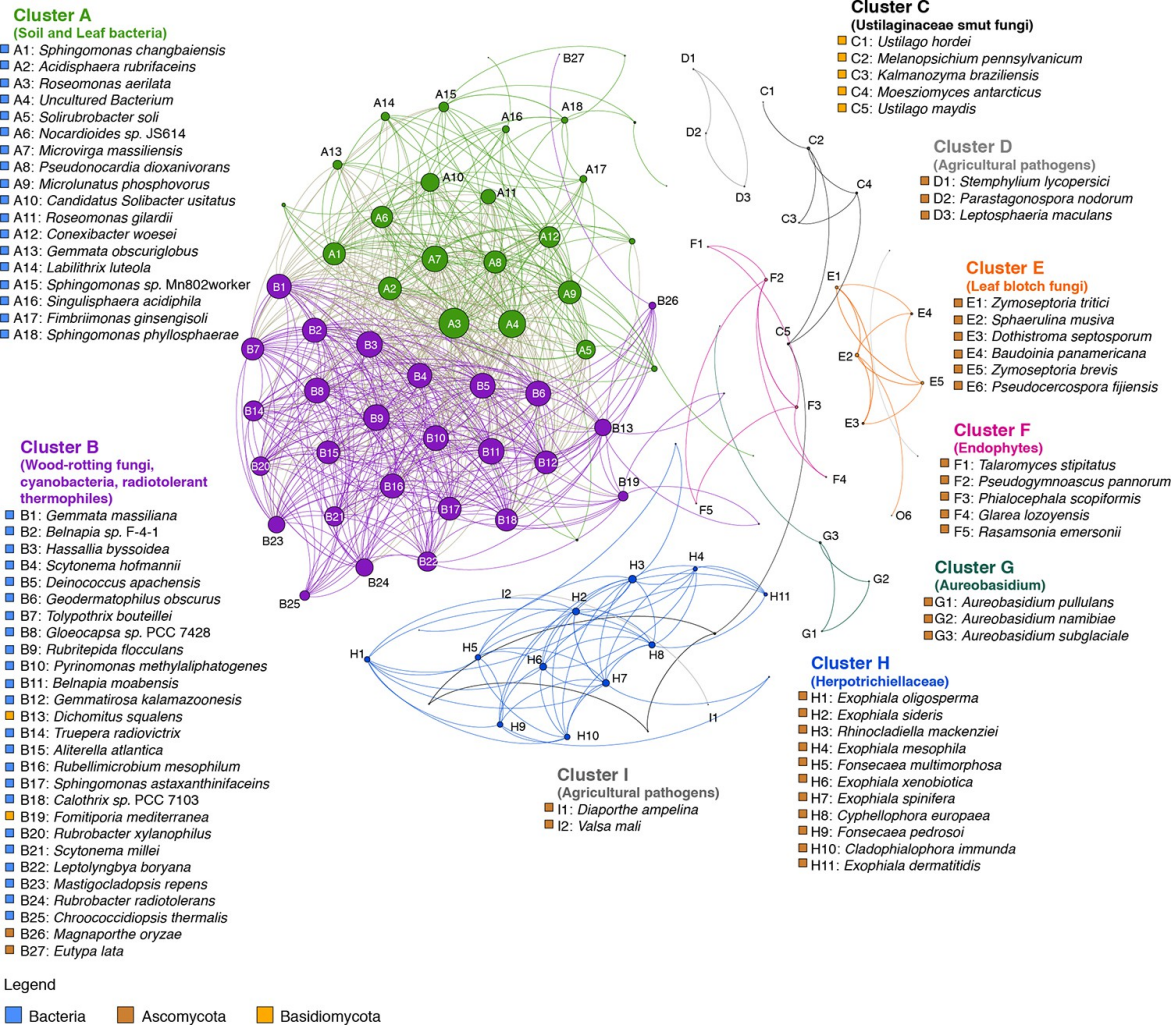
Co-occurrence network analysis of microorganisms on the adaxial and abaxial leaf surfaces. Of the nine clusters identified, two are highly interconnected (clusters A and B). Microorganisms in both clusters are highly abundant on the adaxial surface but are significantly reduced on the abaxial surface. Cluster A is composed of soil and leaf bacteria while cluster B comprises of wood-rotting fungi, photosynthetic cyanobacteria, thermophilic and radiation-tolerant bacteria. The nodes were selected with a cut-off of Spearman rank correlation coefficient of 0.8 and *p*-value of 0.01.

Our co-occurrence network analysis revealed interesting insights of microbial taxa present on the leaf surface. The modularity class algorithm resulted in a total of nine clusters (Fig 3 and S8 Fig). Of these, clusters A and B have highly interconnected nodes. Cluster A is mainly composed of soil and leaf bacteria while the cluster B is comprised of wood-rotting fungi, photosynthetic cyanobacteria, thermophilic and radiation-tolerant bacteria (S9 and S10 Figs). Detailed analyses of the species nodes within these two clusters showed that they are present on the adaxial surface but are extremely reduced on the abaxial surface. Based on the pattern of co-occurrence, we also obtained other clusters that are grouped according to commonalities in phytopathogenic characteristics and phylogeny. For example, cluster E consists of a group of fungi that cause leaf blotch disease [47–49] (S11 Fig), cluster H comprises a group of anamorphic saprotrophs fungi that live on decaying wood and leaves [50] and belong to the Herpotrichiellaceae family (S12 Fig). Cluster C contains a group of phytopathogenic smut fungi that belong to the Ustilaginaceae family [51] (S13 Fig), while cluster G comprises of the genera *Aureobasidium* that is associated with leaves in ecological studies [52, 53] (S14 Fig). Cluster F consists of a group of endophytic fungi [54–56] (S15 Fig) and clusters I and D correspond to agricultural pathogens of apple, wheat, brassica and tomatoes [57–60] (S16 and S17 Figs).

**Fig 4:**
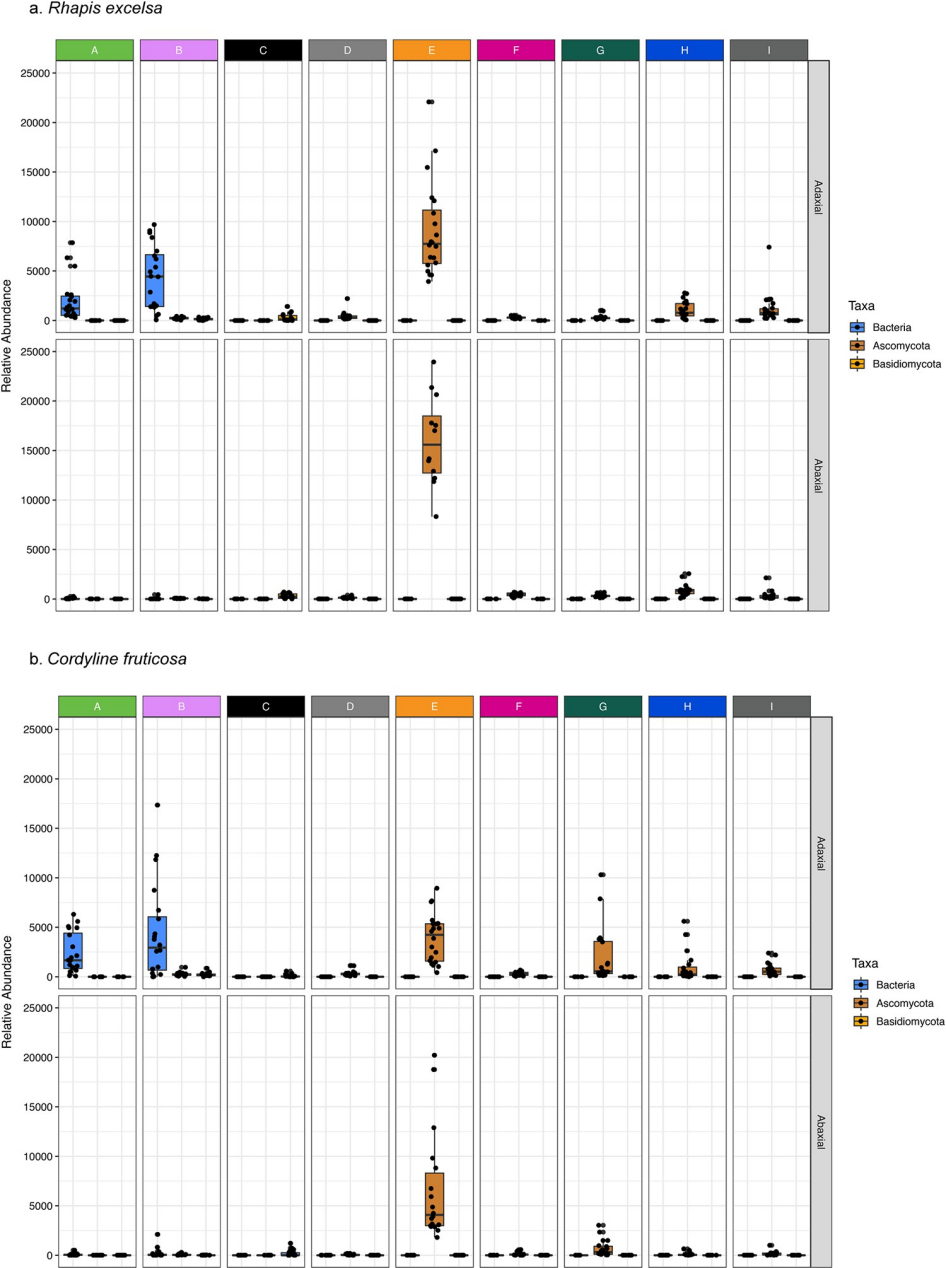
Relative abundance of each microbial co-occurrence cluster. Comparison of the normalised read counts among the nine microbial clusters (A to I) revealed that the orange cluster, composed of leaf blotch fungi, dominates the leaf surfaces of (a) *R*. *excelsa* and (b) *C*. *fruticosa*. The leaf blotch fungi were twice as abundant on the abaxial surface compared to the adaxial counterpart. The dense clusters A and B network observed in Fig 3 suggest an interaction among the group of leaf, soil, and radio-tolerant bacteria on the adaxial surface. These results further detail the reduction in microbial diversity on the abaxial surface.

We compared the relative abundance of Bacteria, Ascomycota, and Basidiomycota among the nine clusters (Fig 4). Firstly, the leaf blotch fungi from cluster E dominate the leaf surfaces, with the abaxial surfaces recording about twice the number of reads assigned to microbial species in cluster E than the adaxial surface. The leaf is a habitat for these ascomycetes in cluster E and it is not surprising to see them with higher abundance on the abaxial surface, where higher densities of stomata and the stomatal pores were observed and likely served as vulnerable ports of entry. Secondly, the highly interconnected network of nodes in the cluster A and B correspond to a group of leaf, soil, and radio-tolerant bacteria, suggesting interactions among themselves as a community on the adaxial surface.

### Leaf surface chemical and physical properties

**Fig 5:**
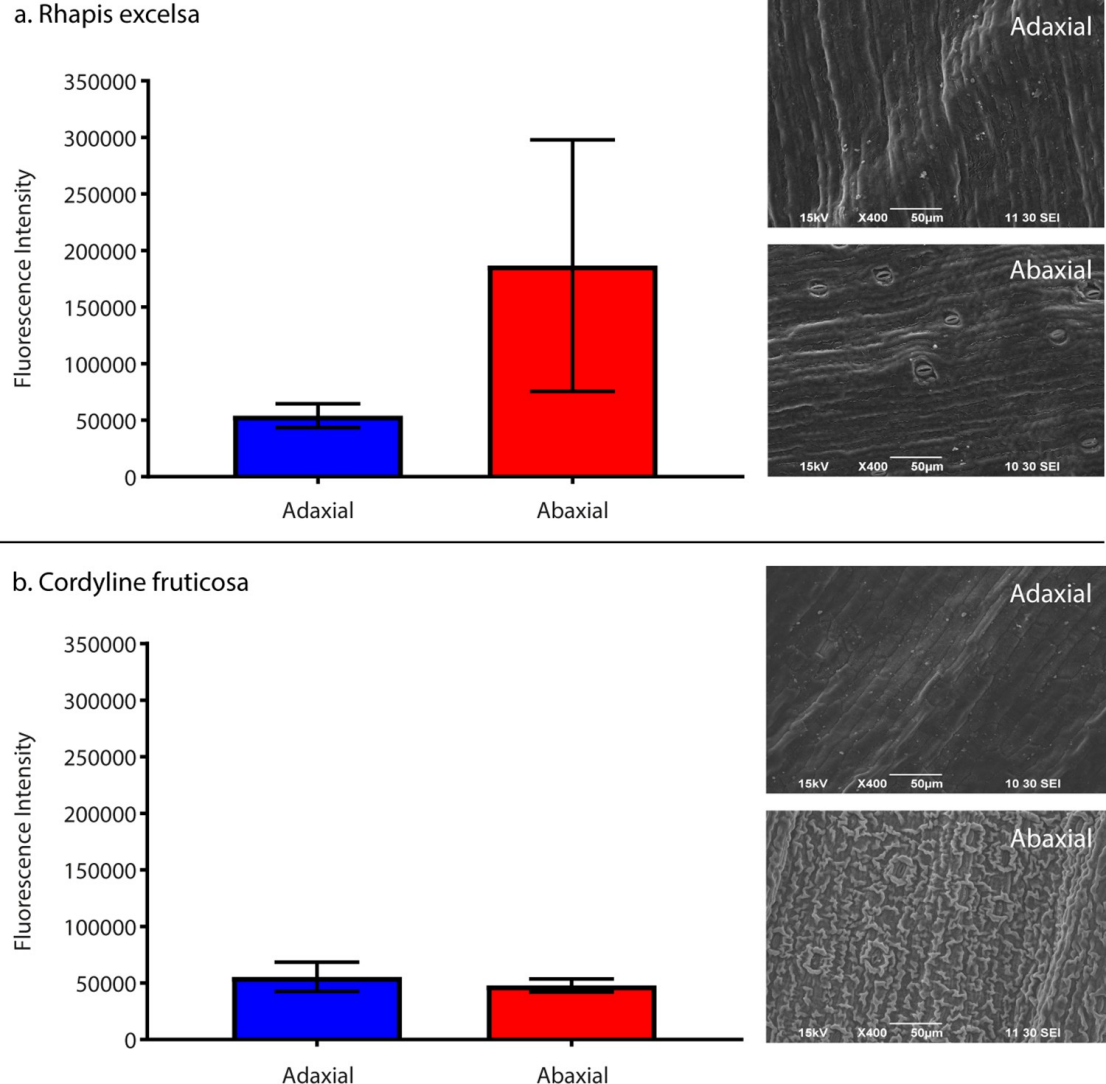
Reactive oxygen species assay of adaxial and abaxial leaf surfaces. A more than 2-fold increase of ROS production was observed on the abaxial surface of (a) *R*. *excelsa*. However, this phenomenon was absent in (b) *C*. *fruticosa*. The fluorescence intensity readings of the ROS assay are listed in S3 Table. The difference in concentration of ROS between adaxial and abaxial surface of *R*. *excelsa* was statistically significant using Welch’s t-test (S5 Table). The photographs were taken by Kenny J.X. Lau.

We tested the swabs of the adaxial and abaxial leaf surfaces in both plants for ROS. Our results showed significantly elevated levels of ROS on the abaxial leaf surface of *R*. *excelsa* across all locations (Fig 5, S18 Fig and S4–S6 Tables). Similar ROS levels, however, were absent in both adaxial and abaxial surface of *C*. *fruticosa* across all locations.

Our SEM images have captured rod-shaped particles that are similar to rod-shaped bacteria and a tube-like structure that resembles a hypha penetrating the stoma (Fig 1). Of the four investigated surfaces, *C*. *fruticosa* had a distinct abaxial leaf surface that had a rough texture with hierarchical structure of 10 μm wrinkled patterns laid with nanometre scales. The abaxial leaf surface has high densities of stomata (Fig 5). Apart from the stomata, there is no structural difference between the adaxial and abaxial leaf surface of *R*. *excelsa*. In contrast, *C*. *fruticosa* has a distinctively patterned abaxial surface as compared to its adaxial counterpart. The abaxial leaf surface has a hierarchical structure that features both micrometre-scale wrinkles and surface projections of nanometre-sized scales.

**Fig 6:**
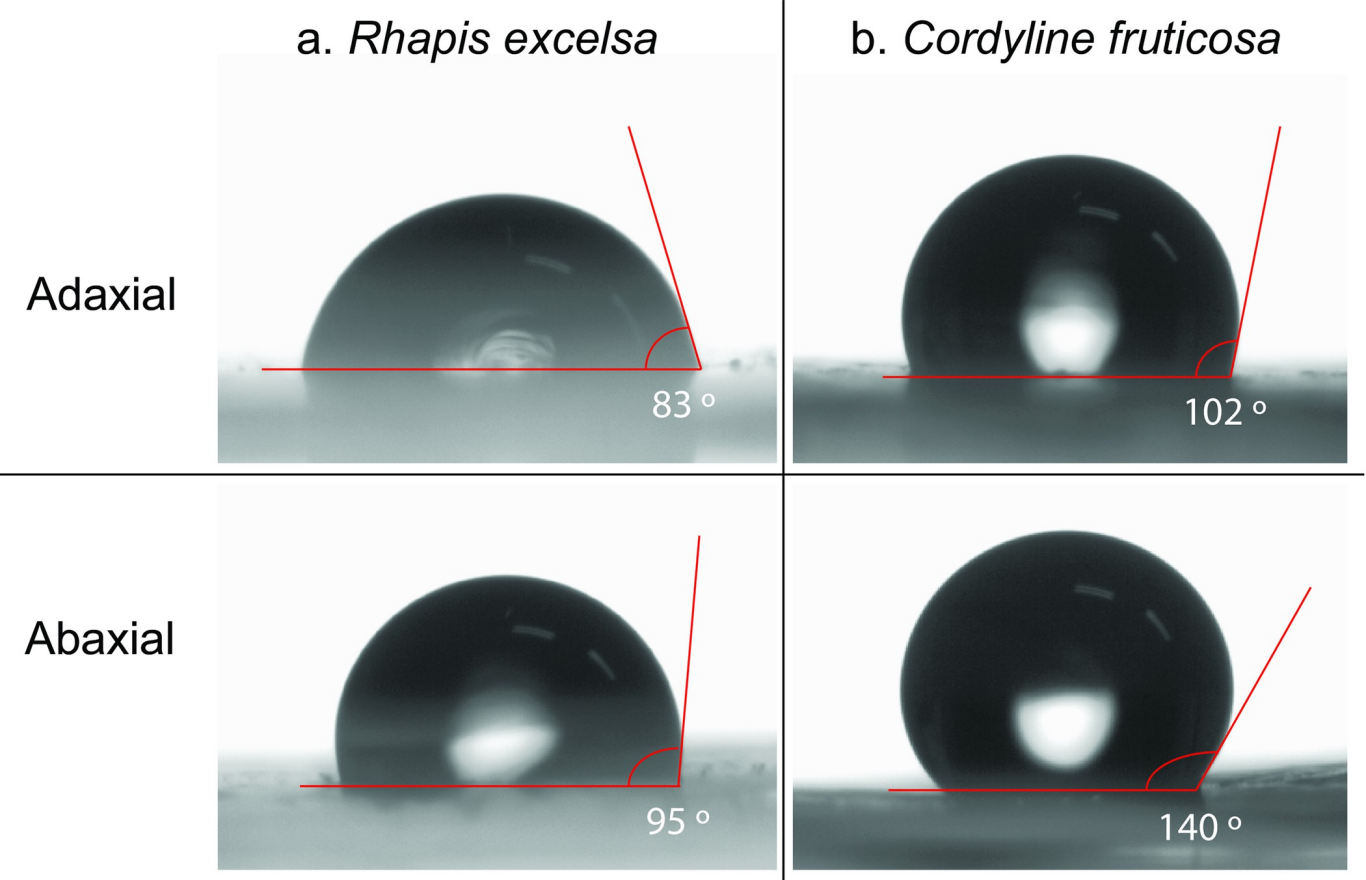
Surface wettability of leaf surfaces. The water contact angle was measured from images captured at orthogonal angle. The adaxial and abaxial leaf surfaces of (a) *R*. *excelsa* have contact angles of 83° and 95°, respectively. The adaxial and abaxial leaf surfaces of (b) *C*. *fruticosa* have contact angles of 102° and 140°, respectively. The abaxial surface is hydrophobic and repels water from its surface. The water droplet images were original images taken by Kenny J.X. Lau using the contact angle meter.

We also tested the hydrophobicity of the leaves by measuring the water contact angle on each leaf surface (Fig 6 and S7 Table). The adaxial leaf surface of *R*. *excelsa* has a contact angle of 83° and is likely to be hydrophilic, while the abaxial surface is 95° and is slightly hydrophobic. *C*. *fruticosa* adaxial leaf surface has a contact angle of 102° and is slightly hydrophobic, while its corresponding abaxial leaf surface has a contact angle of 140°, which is very hydrophobic. We further tested this hydrophobic effect by spreading a 10 μl water droplet on each leaf surfaces with a micropipette tip. Out of the four, only the water droplet on the abaxial surface of *C*. *fruticosa* remained spherical and rolled across the surface (S1–S4 Videos).

## Discussion

### Potential mechanisms that shape leaf microbial community structure

We repeatedly observed a reduction of bacteria on the abaxial leaf surface at different locations, suggesting that this reduction phenomenon is not due to a location specific effect. Our results also reveal that the adaxial and abaxial leaf surfaces of the two plants are structurally and chemically different. There are more stomatal openings on the abaxial surface that could pose as vulnerable ports of entry and it is likely that the plants have evolved adaptations to defend against potential threats.

It is likely that *R*. *excelsa* employ a reactive chemical defence strategy by secreting ROS on its abaxial surface. Entering the plant host tissue is the first critical step for pathogens. Phytopathogenic fungi use their hyphae to penetrate leaf surfaces while bacteria invade into the host tissue through natural openings such as lenticels, stomata, hydathodes, lateral roots and wounds [61–64]. A mechanism has been described for *Arabidopsis thaliana* by which the flagellin receptor, encoded by the gene *fls2*, detects the flagellin epitope, FLG22, of motile bacteria [65]. When *fls2* is activated, it triggers the production of ethylene, ROS, and cell wall reinforcement to inhibit bacterial growth. In a separate study, stomata closure was observed in *Arabidopsis* leaves when live bacteria, such as *Pseudomonas syringae* and *Escherichia coli* O157:H7, were spread onto the leaf surface [11]. Interestingly, the plant responded to small molecules of *FLG22* peptide and purified lipopolysaccharides even in the absence of bacteria. These findings led to the elucidation of the microbial-associated molecular patterns (MAMP)-trigger immunity (MTI) model. MAMPs are epitopes of phytopathogens that may include molecules such as flagellin and lipopolysaccharides from bacteria and chitosan and ergosterol from fungi [66, 67]. Our results suggest that *R*. *excelsa* deploys the MTI defence mechanism to guard its stomata openings on the underside of the leaves.

We next asked the question why fungi, unlike bacteria, are less affected than bacteria by the host defence response (Fig 2, S3 and S4 Figs). There are two broad classes of phytopathogenic fungi: (i) biotrophic that lives on a living host and (ii) necrotrophic that live on dead plant tissues. Nevertheless, some fungi are classified as hemibiotrophic with an initial biotrophic phase that later transforms into a necrotrophic phase upon infection. Most of the fungal species found on both *R*. *excelsa* and *C*. *fruticosa* (S4 Fig) are hemibiotrophic phytopathogens that belong to the classes Dothideomycetes and Sordariomycetes and in the phylum Ascomycota. The top three fungal species in S4 Fig cause leaf spotting. The species *Dothistroma septosporum* causes red band in leaves [68], *Pseudocerospora fijiensis* causes Sigatoka disease in banana leaves [69] and *Colletotrichum gloeosporiodes* are associated with Anthracnose disease in mangoes [70]. *P*. *fijiensis* first establishes itself as an epiphyte growing on the leaf surface and eventually extends its hyphae penetrating through the stomata and into the leaf tissue [71]. Furthermore, its hyphae can produce catalase and superoxide dismutase that allow it to evade the oxidative burst response [72]. *Zymoseptoria tritici*, on the other hand, takes advantage of the oxidative burst response by secreting more ROS to induce plant cell death allowing it to enter the necrotising tissue causing infection [73]. It is likely that the leaf blotch fungi are more resistant to ROS and can colonise the abaxial surface. Thus, we hypothesised that the leaf blotch fungi first invade into the host via the stomatal pores and the plant respond by releasing ROS to eliminate the pathogen. The fungi are immune to ROS and this becomes a positive feedback loop, triggering the plant to produce even higher concentrations of ROS on the abaxial surface. As a result, the abaxial surface becomes toxic to bacteria. This hypothesis explains the reduction of bacterial load seen in *R*. *excelsa* and in contrast, the two-fold increase in leaf blotch fungi on the abaxial side.

Unlike *R*. *excelsa*, *C*. *fruticosa* has a hydrophobic abaxial leaf surface that repels water. Such hydrophobic surfaces wash pathogenic microorganisms away and limit water availability to bacterial cells attempting to form communities on the leaf surface [74]. The hydrophobic defence approach appears more effective strategy in minimising microbial growth when we compared between the two plants. The relative abundance of leaf blotch fungi in *C*. *fruticosa* was half that of *R*. *excelsa* (Fig 4). Despite the low water availability, these leaf blotch fungi remain persistent and continue to colonise the leaves but at a lower abundance. Both oxidative and hydrophobic stress defence strategies result in the low microbial diversity on the abaxial surface.

By combining metagenomics, SEM, chemical and physical properties, our analyses enabled us to establish the above-mentioned comprehensive mechanistic model, which could explain the microbial community structures of leaf surfaces. Our functional analysis using KEGG databases show that the adaxial surfaces of both Rhapis and Cordyline plants harbour a higher DNA read count of gene categories for cell growth, motility, replication, and repair-related activities as opposed to the abaxial surface (S8 Fig). A further study that has also demonstrated the localisation of bacteria on the leaf surface is non-random and highly dependent on leaf microscopic features used live-imaging to demonstrate host interactions with the resident microflora [75]. Further functional tests will be needed to determine the exact underlying molecular mechanisms in both plant systems.

## Conclusions

This study reveals the microbiomes of the adaxial and abaxial leaf surfaces using metagenomic shotgun sequencing on two plant species. A strong reduction in bacterial load and a moderate reduction in fungal load was observed on the abaxial surface compared to the adaxial counterpart. Our co-occurrence analysis further reveals that the abaxial surface is dominated by leaf blotch fungi. In addition, our ROS results suggest that of the two plants, only *R*. *excelsa* releases ROS on the abaxial surface. Based on the literature, leaf blotch fungi are resistant to ROS, but bacteria do not persist in this environment. This is in line with our hypothesis that the plant produces high concentration of ROS in a positive feedback response to eliminate the leaf blotch fungi. In contrast, *C*. *fruticosa* showed no ROS activity but our SEM images captured structural details such as scales and wrinkles imparting hydrophobicity on abaxial surface. This suggests that *C*. *fruticosa* uses a physical defence mechanism to limit the availability of water to minimise microbial growth. This work has improved our understanding that the abaxial leaf surface is distinct from the adaxial surface and that the reduced microbial diversity is possibly a result of plant-microbe interactions at the microscopic level.

## Supporting information

S1 Fig Flowchart of processing pipeline from sampling, DNA extraction, metagenomic shotgun sequencing to bioinformatic data analysis. *Rhapis excelsa* (n = 40) and *Cordyline fruticosa* (n = 40) leaf swabs were collected in Qiagen PowerWater bead tubes until DNA extraction. Samples were sequenced in a multiplexed run, generating a total of 522,825,286 reads for both plant species (S2 Table).
(PDF)

S2 Fig Rarefaction curve of leaf metagenomes from *Rhapis* and *Cordyline* plants. The rarefaction curve shows that for all samples in this study, about 15000 reads is sufficient to reach saturation in the number of taxa assigned on the leaves of the taxonomic tree in MEGAN.
(PDF)

S3 Fig Top 40 bacterial species on the adaxial and abaxial leaf surface. The sun and moon symbols represent the time of sampling while letters refer to the sampling sites. Significant reduction in bacterial read counts was observed in both (a) *Rhapis excelsa* and (b) *Cordyline fruticosa* despite the location and time of day.
(PDF)

S4 Fig Top 40 fungal species on the adaxial and abaxial leaf surface. The top fungal species in both (a) *Rhapis excelsa* and (b) *Cordyline fruticosa* leaves are hemibiotrophic phytopathogens that belong to the classes Dothideomycetes and Sordariomycetes and in the phylum Ascomycota.
(PDF)

S5 Fig Principal coordinate analysis plot of microbiomes on the adaxial and abaxial leaf surface. Two distinct clusters were observed with segregation between adaxial and abaxial leaf surface microbiomes along the first principal coordinate axis in (a) *Rhapis excelsa* at 42.9% of variation explained and (b) *Cordyline fruticosa* at 36.2% of variation explained. ANOSIM showed strong segregation between adaxial and abaxial groups with R-statistic of 0.7322 and 0.717 for *R*. *excelsa* and *C*. *fruticosa* respectively.
(PDF)

S6 Fig Principal coordinate (PCoA) plots of metagenomes of both adaxial and abaxial leaf surfaces from both plants overlaid with spatiotemporal factor information. (a) Two 6 distinct clusters of adaxial and abaxial leaf surfaces were observed along PCo1 with 33.1% of 7 variance explained, the same samples in (b) were coloured by time of sampling and (c) by 8 locations. No clear clustering was observed between day and night and locations. The samples 9 in (d) showed that plant species explain the spread along PCo2 axis.
(PDF)

S7 Fig Metagenomic reads mapped to functional gene pathways in KEGG database. The 21 adaxial leaf surface has more reads with higher z-scores that are mapped to cell growth, motility, 22 replication and repair-related activities as compared to the abaxial leaf surface observed in both 23 (a) *Rhapis excelsa* and (b) *Cordyline fruticosa*.
(PDF)

S8 Fig Relative proportions of each co-occurrence cluster. Based on the co-occurrence plot in Fig 6, we expected that the dense network between cluster A and E would be the most dominant 15 microbial component. However, it was cluster E, the leaf blotch fungi, that was more prevalent 16 in the leaves of both plants.
(PDF)

S9 Fig Microorganisms in cluster A. Cluster A consists of only bacterial species. Reduction in the 27 relative abundances of bacteria was observed on the abaxial leaf surface as compared to the 28 adaxial surface.
(PDF)

S10 Fig Microorganisms in cluster B. Cluster B consists of mostly bacteria and four fungal species. 32 Like cluster A, a reduction in the relative abundances of microbes was observed on the abaxial 33 leaf surface as compared to the adaxial surface.
(PDF)

S11 Fig Microorganisms in cluster E. Cluster E is composed of leaf blotch fungal species. Their 37 relative abundances are the highest among all other clusters. Furthermore, the abaxial leaf 38 surface had approximately twice the number of microbes as compared to the adaxial surface.
(PDF)

S12 Fig Microorganisms in cluster H. Cluster H has fungi that belong to the Herpotrichiellaceae 42 family. They were more abundant on *R*. *excelsa* leaves than *C*. *fruticosa* leaves.
(PDF)

S13 Fig Microorganisms in cluster C. Cluster C consists of fungi from the phyla, Basidiomycota. 46 Their frequencies were sporadic and varied from one location to another.
(PDF)

S14 Fig Microorganisms in cluster G. *Aureobasidium spp*. is ten times more prevalent on *C*. *fruticosa* 50 leaves than *R*. *excelsa* leaves.
(PDF)

S15 Fig Microorganisms in cluster F. Cluster F consists of fungi that are mainly leaf endophytes, 54 with about 10–200 reads. They seemed to be more abundant in *R*. *excelsa* than *C*. *fruticosa*.
(PDF)

S16 Fig Microorganisms in cluster I. Cluster I consists of agricultural pathogens. They were slightly 58 more abundant on the adaxial surface than the abaxial and were more prevalent on *R*. *excelsa*.
(PDF)

S17 Fig Microorganisms in cluster D. Cluster D comprises of agricultural pathogens and they were 62 slightly more abundant on the adaxial leaf surface.
(PDF)

S18 Fig Reactive oxygen assay of adaxial and abaxial leaf surfaces. More than 2-fold increase 66 of ROS production on the abaxial surface of (a) *R*. *excelsa*. However, this phenomenon was 67 absent in (b) *C*. *fruticosa*. The fluorescence intensity readings of the ROS assay can be found 68 in S3 and S4 Tables.
(PDF)

S1 Table Pairwise relative distances in metres between sampling sites. (PDF)

S2 Table Average number of reads generated for the adaxial and abaxial leaf surfaces at various locations. (PDF)

S3 Table Mann Whitney test of *Chao-1* species richness estimates index between adaxial and abaxial leaf surface. (PDF)

S4 Table Reactive oxygen species assay. (PDF)

S5 Table Reactive oxygen species assay. (PDF)

S6 Table F-test and t-test of reactive oxygen species concentration between adaxial and abaxial leaf surface. (PDF)

S7 Table Water contact angle measurements. (PDF)

S1 Video Hydrophobicity test on *Rhapis excelsa*—adaxial leaf. (MP4)

S2 Video Hydrophobicity test on *Rhapis excelsa*—abaxial leaf. (MP4)

S3 Video Hydrophobicity test on *Cordyline fruticosa*—adaxial leaf. (MP4)

S4 Video Hydrophobicity test on *Cordyline fruticosa*—abaxial leaf. (MP4)
