# Supplementary material for: Structure vs. chemistry: Alternate mechanisms for controlling leaf microbiomes
Source: PLoS One. 2023 Mar 21;18(3):e0275734. doi: 10.1371/journal.pone.0275734 (PMC10030040; doi:10.1371/journal.pone.0275734)
Supplement: S1 Fig — Rhapis excelsa (n = 40) and Cordyline fruticosa (n = 40) leaf swabs were collected in Qiagen PowerWater bead tubes until DNA extraction. Samples were sequenced in a multiplexed run, generating a total of 522,825,286 reads for both plant species (S2 Table). (PDF) [file pone.0275734.s001.pdf]

S1 Fig

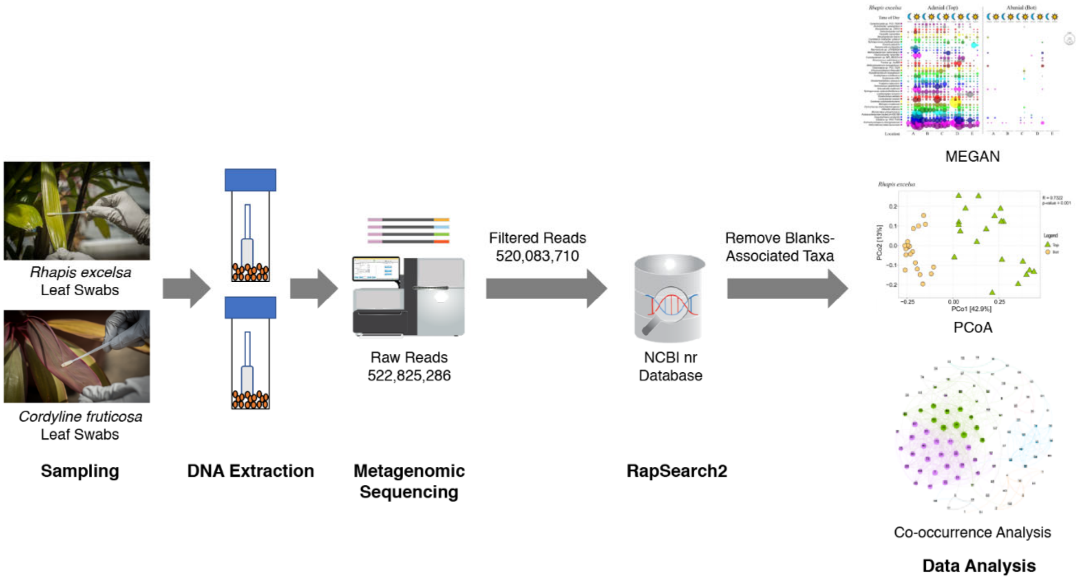

Flowchart of processing pipeline from sampling, DNA extraction, metagenomic shotgun sequencing to bioinformatic data analysis. *Rhapis excelsa* (n = 40) and *Cordyline fruticosa* (n = 40) leaf swabs were collected in Qiagen PowerWater bead tubes until DNA extraction. Samples were sequenced in a multiplexed run, generating a total of 522,825,286 reads for both plant species (Table S2).
