# Supplementary material for: Structure vs. chemistry: Alternate mechanisms for controlling leaf microbiomes
Source: PLoS One. 2023 Mar 21;18(3):e0275734. doi: 10.1371/journal.pone.0275734 (PMC10030040; doi:10.1371/journal.pone.0275734)
Supplement: S2 Fig — The rarefaction curve shows that for all samples in this study, about 15000 reads is sufficient to reach saturation in the number of taxa assigned on the leaves of the taxonomic tree in MEGAN. (PDF) [file pone.0275734.s002.pdf]

S2 Fig

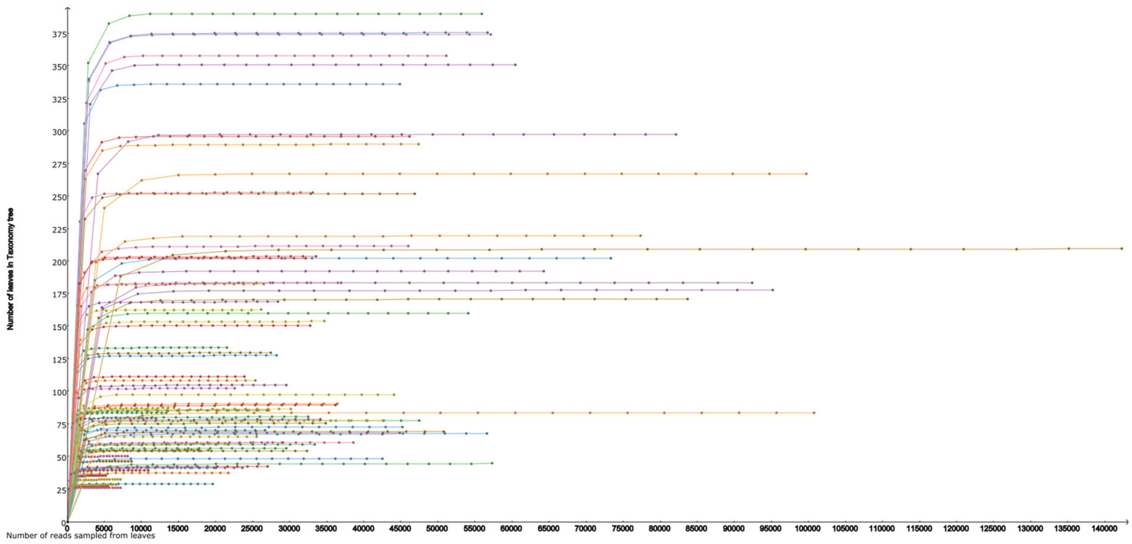

Rarefaction curve of leaf metagenomes from *Rhapis* and *Cordyline* plants. The rarefaction curve shows that for all samples in this study, about 15000 reads is sufficient to reach saturation in the number of taxa assigned on the leaves of the taxonomic tree in MEGAN.
