# Supplementary material for: Structure vs. chemistry: Alternate mechanisms for controlling leaf microbiomes
Source: PLoS One. 2023 Mar 21;18(3):e0275734. doi: 10.1371/journal.pone.0275734 (PMC10030040; doi:10.1371/journal.pone.0275734)
Supplement: S3 Fig — The sun and moon symbols represent the time of sampling while letters refer to the sampling sites. Significant reduction in bacterial read counts was observed in both (a) Rhapis excelsa and (b) Cordyline fruticosa despite the location and time of day. (PDF) [file pone.0275734.s003.pdf]

Fig S3

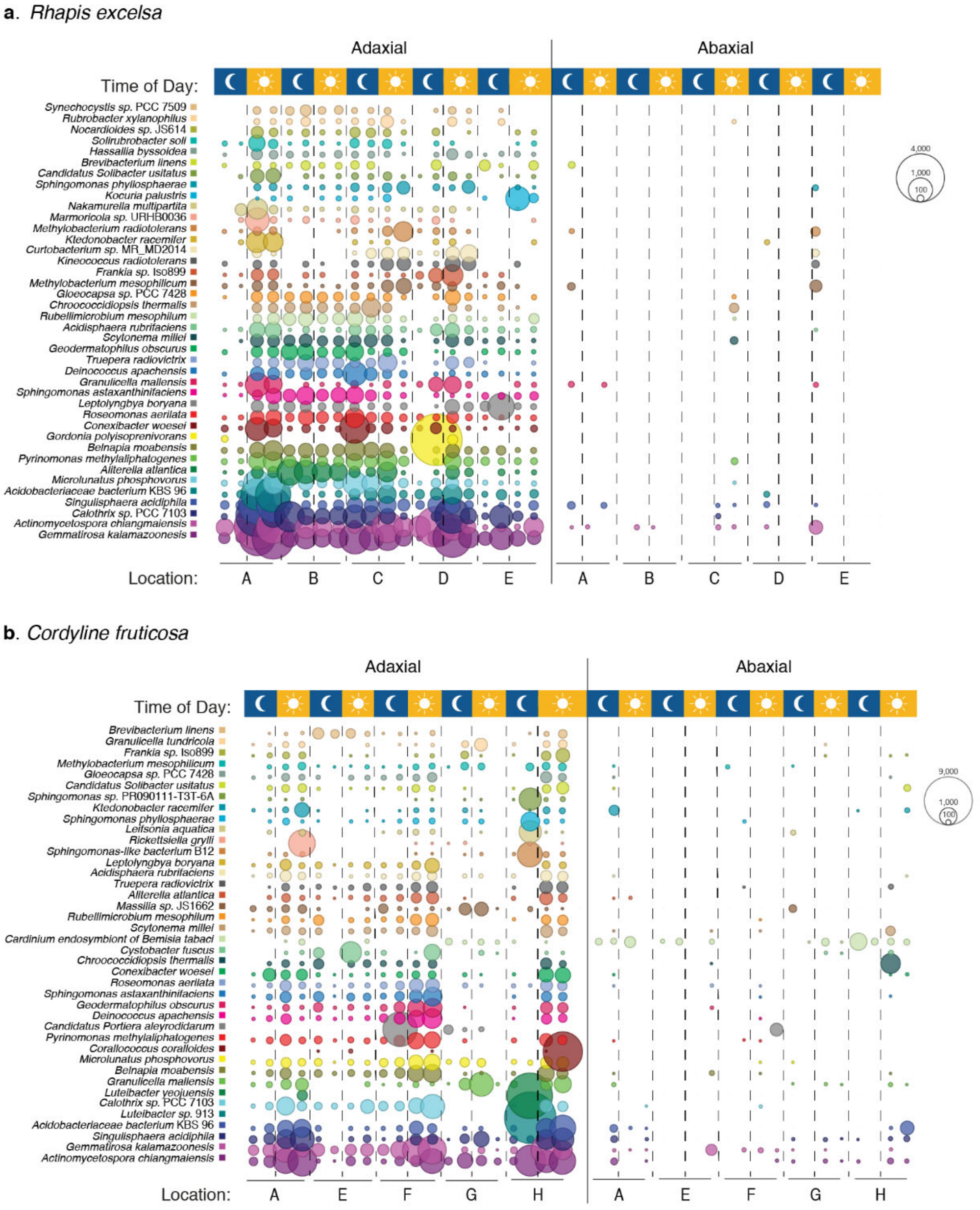

Top 40 bacterial species on the adaxial and abaxial leaf surface. The sun and moon symbols represent the time of sampling while letters refer to the sampling sites. Significant reduction in bacterial read counts was observed in both (a) *Rhapis excelsa* and (b) *Cordyline fruticosa* despite the location and time of day.
