# Supplementary figures and images for: Structure vs. chemistry: Alternate mechanisms for controlling leaf microbiomes
Source: PLoS One. 2023 Mar 21;18(3):e0275734. doi: 10.1371/journal.pone.0275734 (PMC10030040; doi:10.1371/journal.pone.0275734)

**a. *Rhapis excelsa***

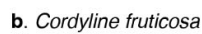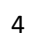

Supplement: S4 Fig — The top fungal species in both (a) Rhapis excelsa and (b) Cordyline fruticosa leaves are hemibiotrophic phytopathogens that belong to the classes Dothideomycetes and Sordariomycetes and in the phylum Ascomycota. (PDF) [file pone.0275734.s004.pdf]
