# Supplementary material for: Structure vs. chemistry: Alternate mechanisms for controlling leaf microbiomes
Source: PLoS One. 2023 Mar 21;18(3):e0275734. doi: 10.1371/journal.pone.0275734 (PMC10030040; doi:10.1371/journal.pone.0275734)
Supplement: S5 Fig — Two distinct clusters were observed with segregation between adaxial and abaxial leaf surface microbiomes along the first principal coordinate axis in (a) Rhapis excelsa at 42.9% of variation explained and (b) Cordyline fruticosa at 36.2% of variation explained. ANOSIM showed strong segregation between adaxial and abaxial groups with R-statistic of 0.7322 and 0.717 for R. excelsa and C. fruticosa respectively. (PDF) [file pone.0275734.s005.pdf]

S5 Fig

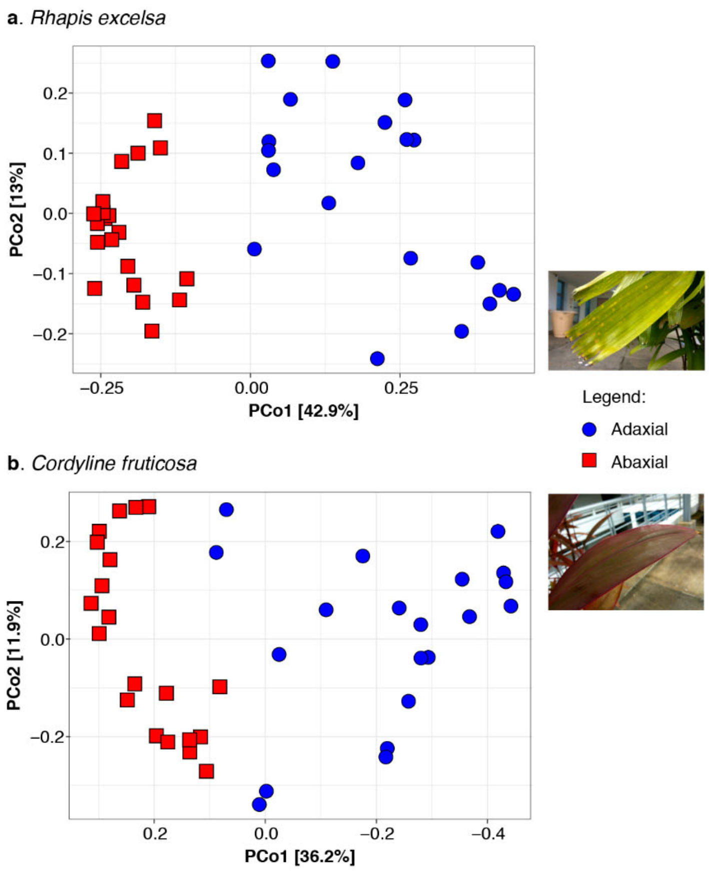

Principal Coordinate Analysis plot of microbiomes on the adaxial and abaxial leaf surface. Two distinct clusters were observed with segregation between adaxial and abaxial leaf surface microbiomes along the first principal coordinate axis in (a) *Rhapis excelsa* at 42.9% of variation explained and (b) *Cordyline fruticosa* at 36.2% of variation explained. ANOSIM showed strong segregation between adaxial and abaxial groups with *R*-statistic of 0.7322 and 0.717 for *R. excelsa* and *C. fruticosa* respectively.
