# Supplementary material for: Structure vs. chemistry: Alternate mechanisms for controlling leaf microbiomes
Source: PLoS One. 2023 Mar 21;18(3):e0275734. doi: 10.1371/journal.pone.0275734 (PMC10030040; doi:10.1371/journal.pone.0275734)
Supplement: S6 Fig — (a) Two 6 distinct clusters of adaxial and abaxial leaf surfaces were observed along PCo1 with 33.1% of 7 variance explained, the same samples in (b) were coloured by time of sampling and (c) by 8 locations. No clear clustering was observed between day and night and locations. The samples 9 in (d) showed that plant species explain the spread along PCo2 axis. (PDF) [file pone.0275734.s006.pdf]

S6 Fig

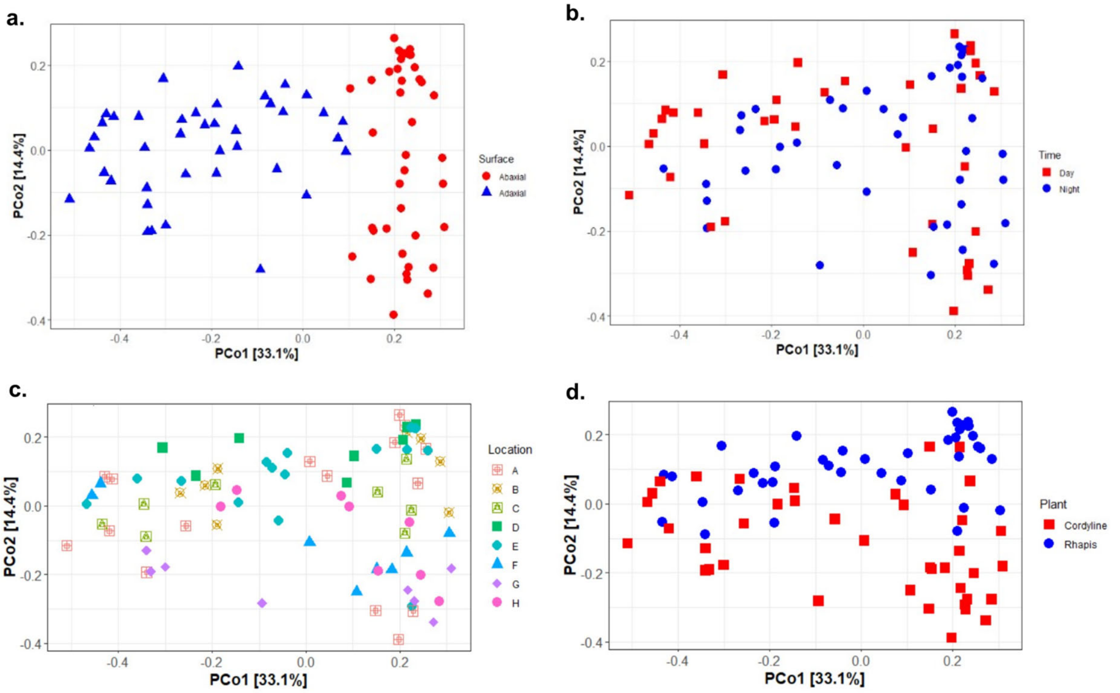

Principal Coordinate (PCoA) plots of metagenomes of both adaxial and abaxial leaf surfaces from both plants overlaid with spatiotemporal factor information. (a) Two distinct clusters of adaxial and abaxial leaf surfaces were observed along PCo1 with 33.1% of variance explained, the same samples in (b) were coloured by time of sampling and (c) by locations. No clear clustering was observed between day and night and locations. The samples in (d) showed that plant species explain the spread along PCo2 axis.
