# Supplementary material for: Structure vs. chemistry: Alternate mechanisms for controlling leaf microbiomes
Source: PLoS One. 2023 Mar 21;18(3):e0275734. doi: 10.1371/journal.pone.0275734 (PMC10030040; doi:10.1371/journal.pone.0275734)
Supplement: S7 Fig — The 21 adaxial leaf surface has more reads with higher z-scores that are mapped to cell growth, motility, 22 replication and repair-related activities as compared to the abaxial leaf surface observed in both 23 (a) Rhapis excelsa and (b) Cordyline fruticosa. (PDF) [file pone.0275734.s007.pdf]

S7 Fig

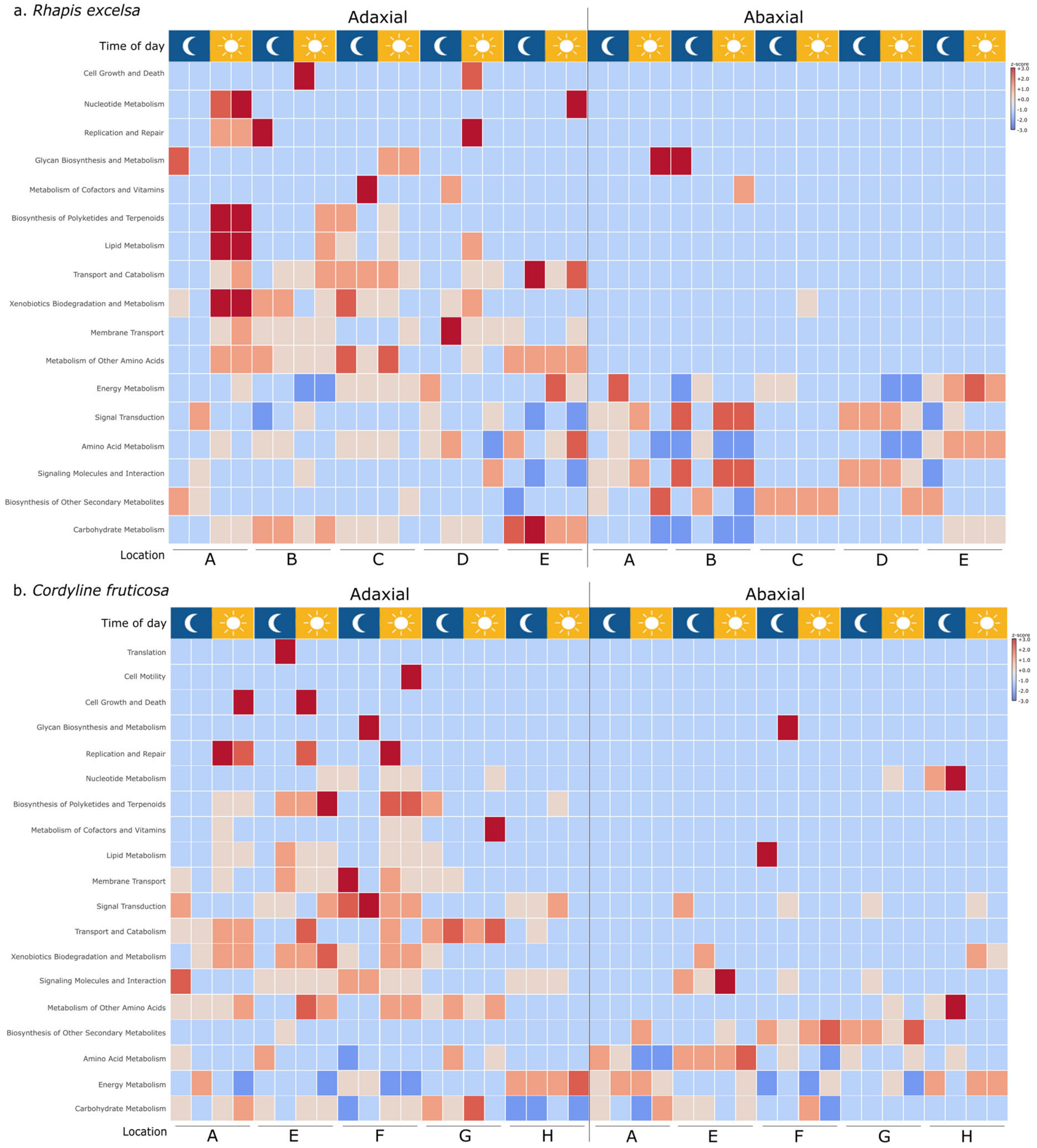

Metagenomic reads mapped to functional gene pathways in the KEGG database. The adaxial leaf surface has more reads with higher z-scores that are mapped to cell growth, motility, replication and repair-related activities as compared to the abaxial leaf surface observed in both (a) *Rhapis excelsa* and (b) *Cordyline fruticosa*.
