# Supplementary material for: Structure vs. chemistry: Alternate mechanisms for controlling leaf microbiomes
Source: PLoS One. 2023 Mar 21;18(3):e0275734. doi: 10.1371/journal.pone.0275734 (PMC10030040; doi:10.1371/journal.pone.0275734)
Supplement: S8 Fig — Based on the co-occurrence plot in Fig 6, we expected that the dense network between cluster A and E would be the most dominant 15 microbial component. However, it was cluster E, the leaf blotch fungi, that was more prevalent 16 in the leaves of both plants. (PDF) [file pone.0275734.s008.pdf]

S8 Fig

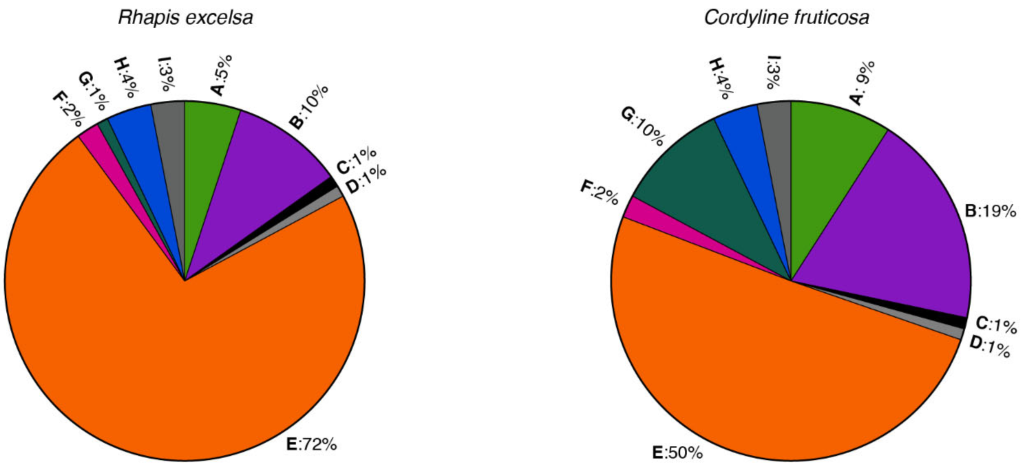

Relative proportions of each co-occurrence cluster. Based on the co-occurrence plot in Fig. 6, we expected that the dense network between cluster A and E would be the most dominant microbial component. However, it was cluster E, the leaf blotch fungi, that was more prevalent in the leaves of both plants.
