# Supplementary material for: Structure vs. chemistry: Alternate mechanisms for controlling leaf microbiomes
Source: PLoS One. 2023 Mar 21;18(3):e0275734. doi: 10.1371/journal.pone.0275734 (PMC10030040; doi:10.1371/journal.pone.0275734)
Supplement: S9 Fig — Cluster A consists of only bacterial species. Reduction in the 27 relative abundances of bacteria was observed on the abaxial leaf surface as compared to the 28 adaxial surface. (PDF) [file pone.0275734.s009.pdf]

S9 Fig

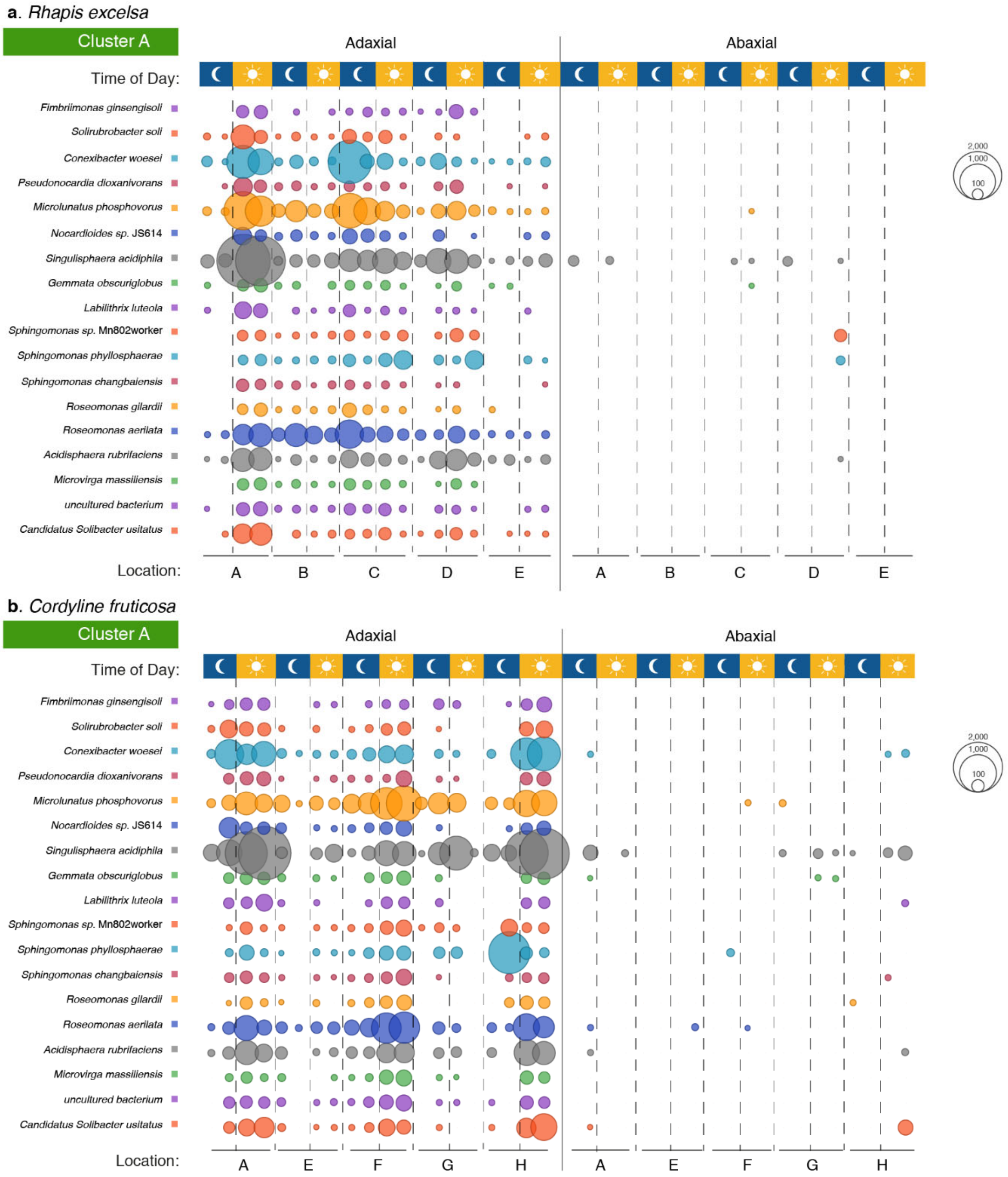

Microorganisms in Cluster A. Cluster A consists of only bacterial species. Reduction in the relative abundances of bacteria was observed on the abaxial leaf surface as compared to the adaxial surface.
