# Supplementary material for: Structure vs. chemistry: Alternate mechanisms for controlling leaf microbiomes
Source: PLoS One. 2023 Mar 21;18(3):e0275734. doi: 10.1371/journal.pone.0275734 (PMC10030040; doi:10.1371/journal.pone.0275734)
Supplement: S10 Fig — Cluster B consists of mostly bacteria and four fungal species. 32 Like cluster A, a reduction in the relative abundances of microbes was observed on the abaxial 33 leaf surface as compared to the adaxial surface. (PDF) [file pone.0275734.s010.pdf]

S10 Fig

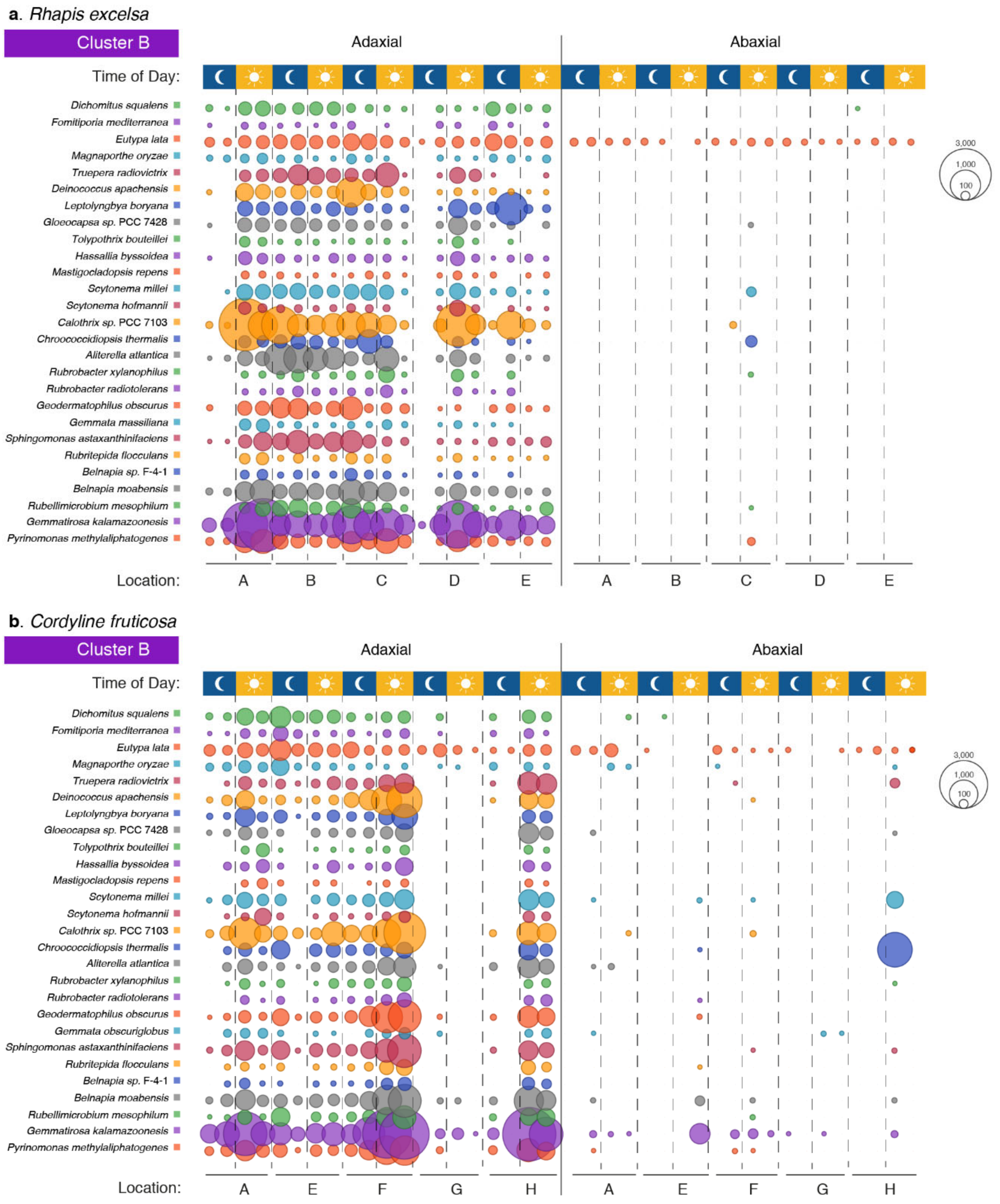

Microorganisms in Cluster B. Cluster B consists of mostly bacteria and four fungal species. Like cluster A, a reduction in the relative abundances of microbes was observed on the abaxial leaf surface as compared to the adaxial surface.
