# Supplementary material for: Structure vs. chemistry: Alternate mechanisms for controlling leaf microbiomes
Source: PLoS One. 2023 Mar 21;18(3):e0275734. doi: 10.1371/journal.pone.0275734 (PMC10030040; doi:10.1371/journal.pone.0275734)
Supplement: S11 Fig — Cluster E is composed of leaf blotch fungal species. Their 37 relative abundances are the highest among all other clusters. Furthermore, the abaxial leaf 38 surface had approximately twice the number of microbes as compared to the adaxial surface. (PDF) [file pone.0275734.s011.pdf]

S11 Fig

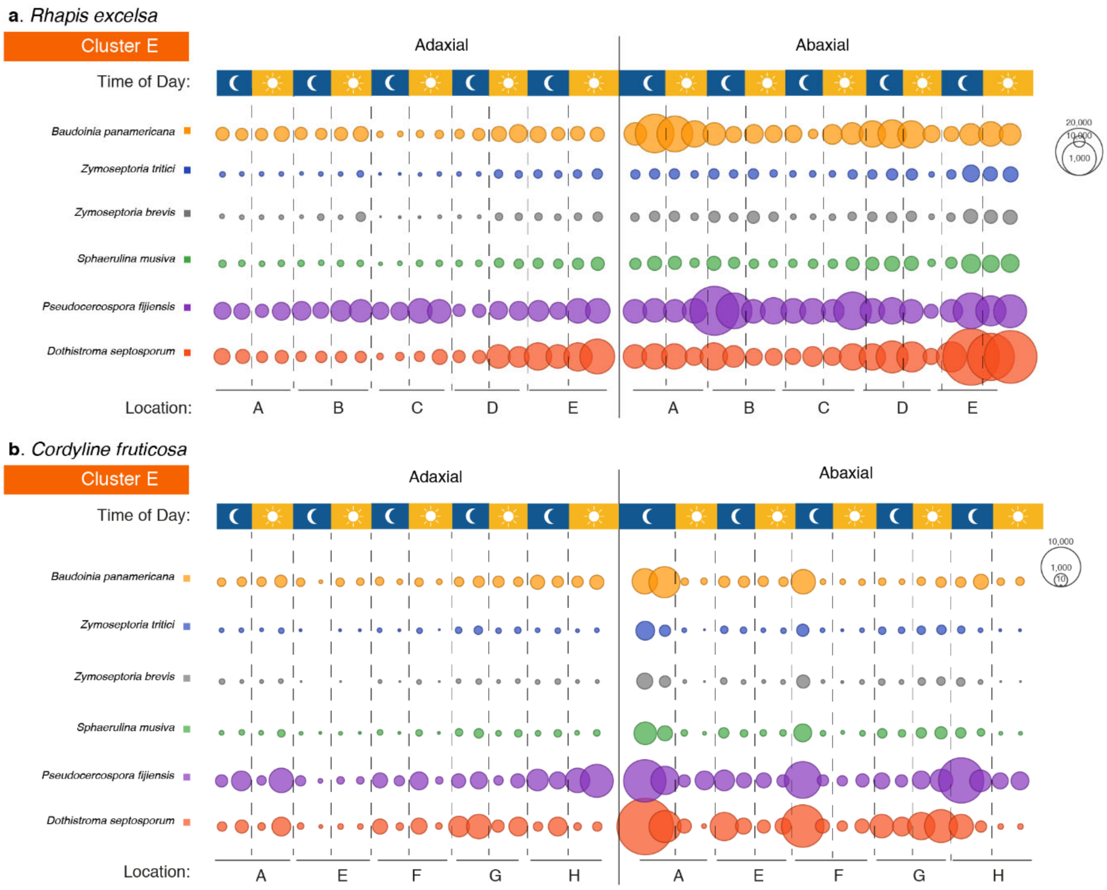

Microorganisms in Cluster E. Cluster E is composed of leaf blotch fungal species. Their relative abundances are the highest among all other clusters. Furthermore, the abaxial leaf surface had approximately twice the number of microbes as compared to the adaxial surface.
