# Supplementary material for: Structure vs. chemistry: Alternate mechanisms for controlling leaf microbiomes
Source: PLoS One. 2023 Mar 21;18(3):e0275734. doi: 10.1371/journal.pone.0275734 (PMC10030040; doi:10.1371/journal.pone.0275734)
Supplement: S12 Fig — Cluster H has fungi that belong to the Herpotrichiellaceae 42 family. They were more abundant on R. excelsa leaves than C. fruticosa leaves. (PDF) [file pone.0275734.s012.pdf]

S12 Fig

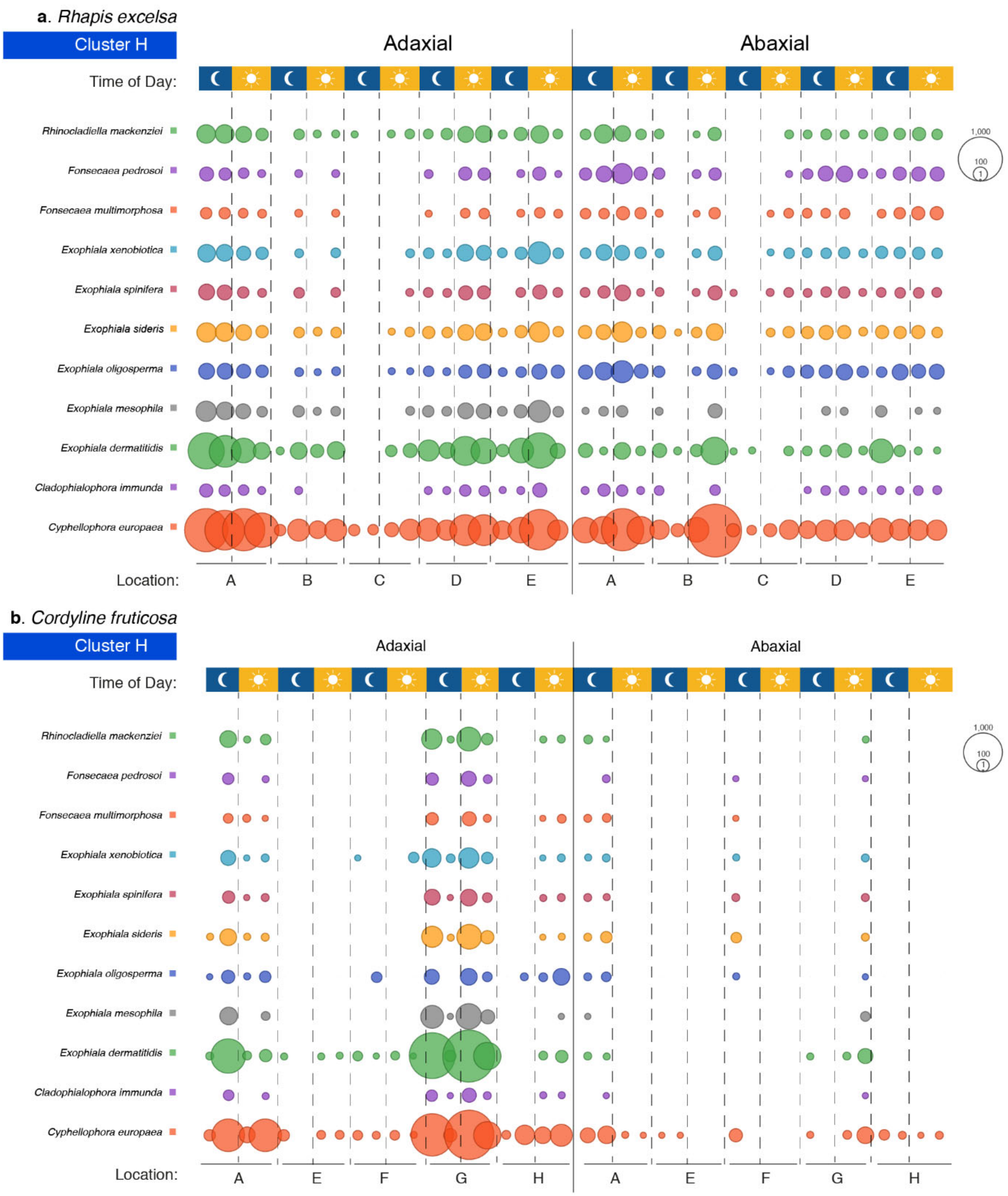

Microorganisms in Cluster H. Cluster H has fungi that belong to the Herpotrichiellaceae family. They were more abundant on *R. excelsa* leaves than *C. fruticosa* leaves.
