# Supplementary material for: Structure vs. chemistry: Alternate mechanisms for controlling leaf microbiomes
Source: PLoS One. 2023 Mar 21;18(3):e0275734. doi: 10.1371/journal.pone.0275734 (PMC10030040; doi:10.1371/journal.pone.0275734)
Supplement: S13 Fig — Cluster C consists of fungi from the phyla, Basidiomycota. 46 Their frequencies were sporadic and varied from one location to another. (PDF) [file pone.0275734.s013.pdf]

43

S13 Fig

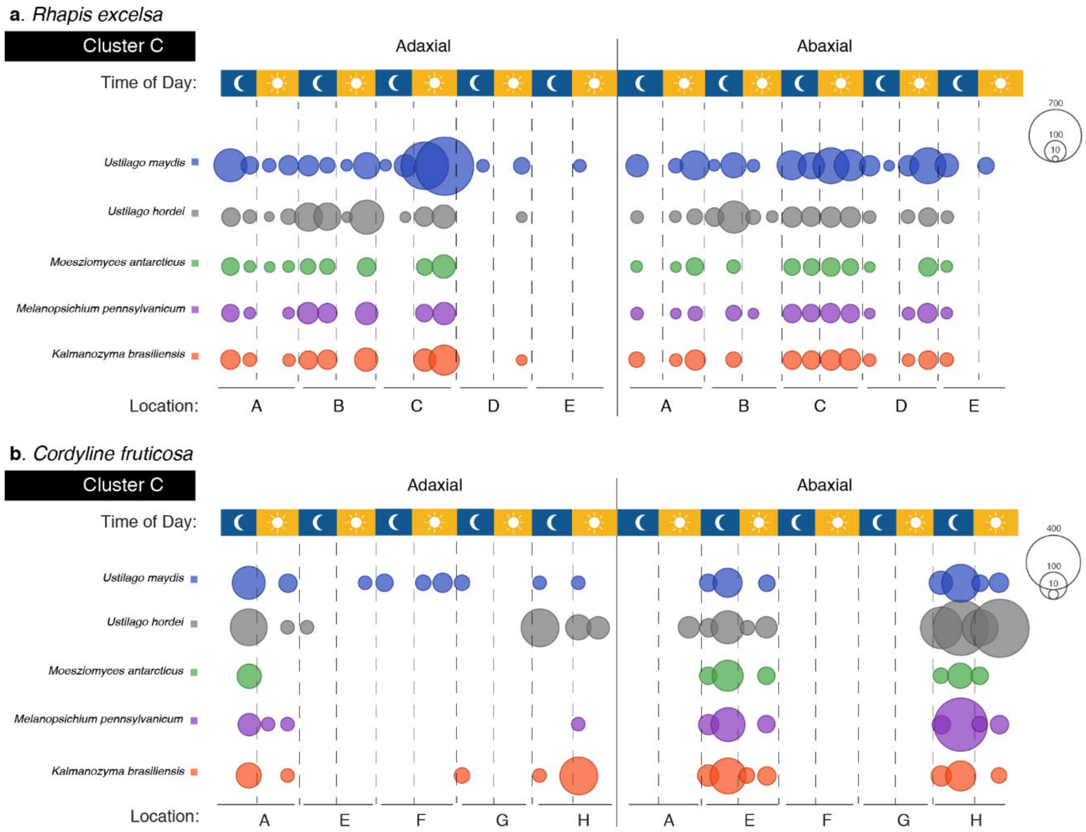

44

45 **Microorganisms in Cluster C.** Cluster C consists of fungi from the phyla, Basidiomycota.

46 Their frequencies were sporadic and varied from one location to another.
