# Supplementary material for: Structure vs. chemistry: Alternate mechanisms for controlling leaf microbiomes
Source: PLoS One. 2023 Mar 21;18(3):e0275734. doi: 10.1371/journal.pone.0275734 (PMC10030040; doi:10.1371/journal.pone.0275734)
Supplement: S14 Fig — Aureobasidium spp. is ten times more prevalent on C. fruticosa 50 leaves than R. excelsa leaves. (PDF) [file pone.0275734.s014.pdf]

S14 Fig

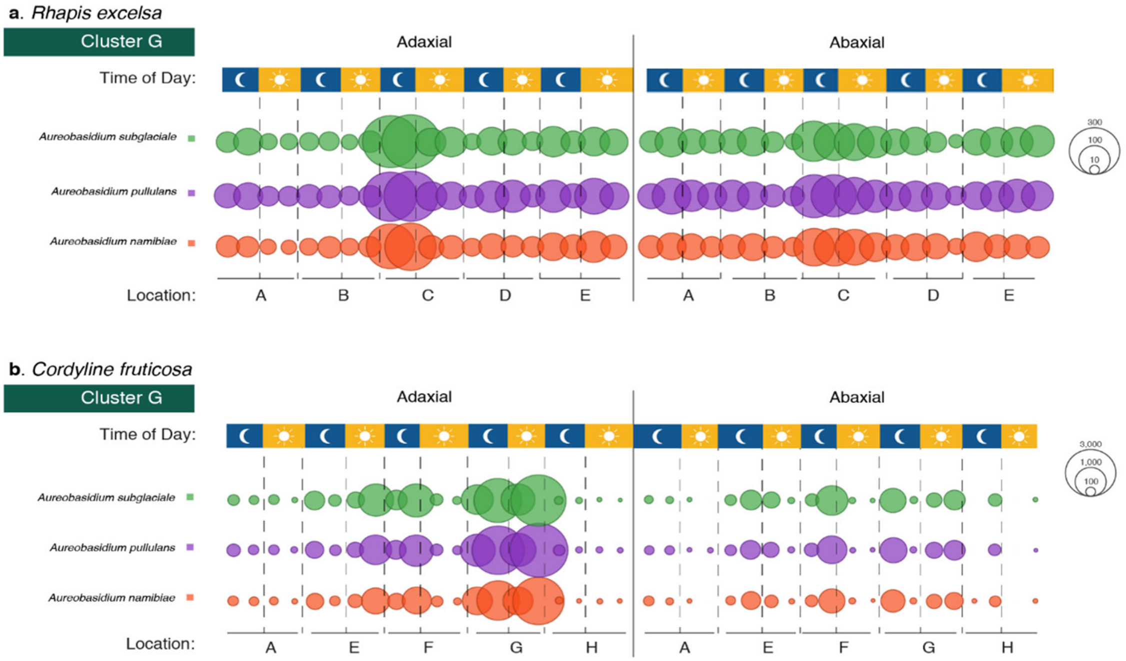

Microorganisms in Cluster G. *Aureobasidium spp.* is ten times more prevalent on *C. fruticosa* leaves than *R. excelsa* leaves.
