# Supplementary material for: Structure vs. chemistry: Alternate mechanisms for controlling leaf microbiomes
Source: PLoS One. 2023 Mar 21;18(3):e0275734. doi: 10.1371/journal.pone.0275734 (PMC10030040; doi:10.1371/journal.pone.0275734)
Supplement: S15 Fig — Cluster F consists of fungi that are mainly leaf endophytes, 54 with about 10–200 reads. They seemed to be more abundant in R. excelsa than C. fruticosa. (PDF) [file pone.0275734.s015.pdf]

S15 Fig

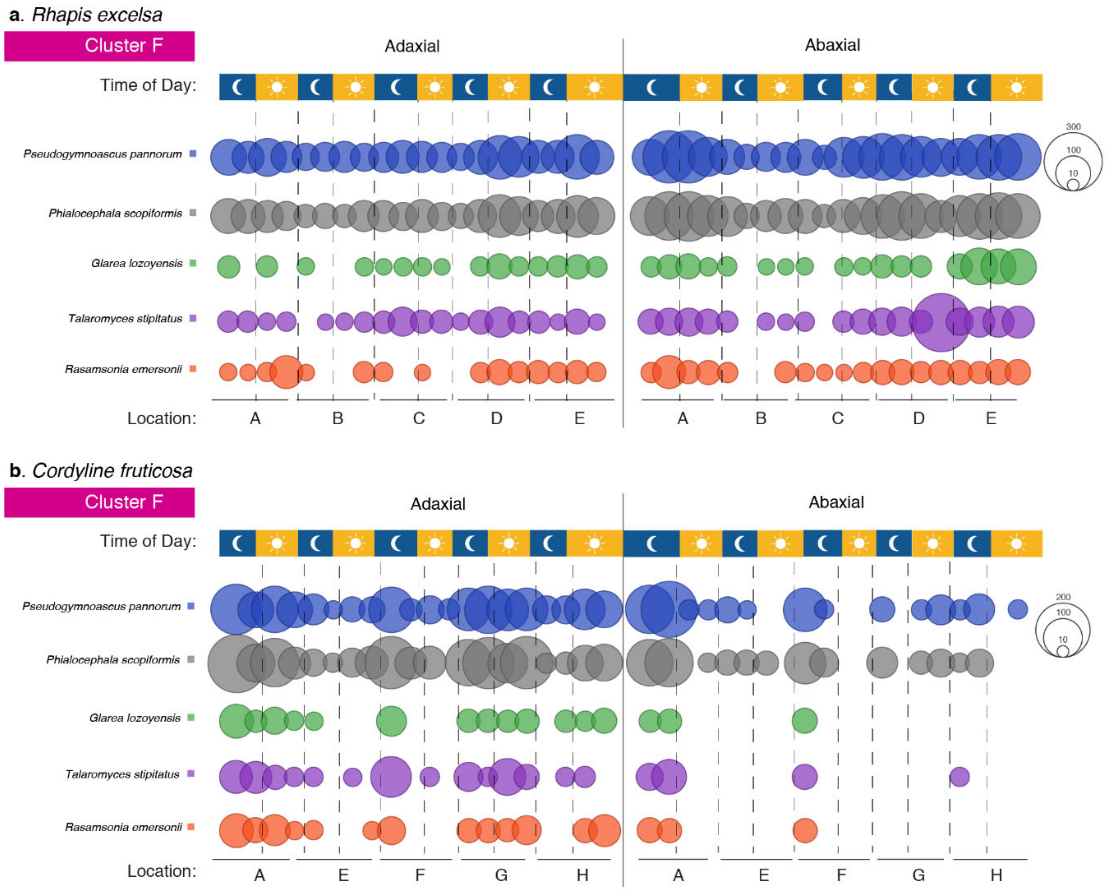

Microorganisms in Cluster F. Cluster F consists of fungi that are mainly leaf endophytes, with about 10-200 reads. They seemed to be more abundant in *R. excelsa* than *C. fruticosa*.
