# Supplementary material for: Structure vs. chemistry: Alternate mechanisms for controlling leaf microbiomes
Source: PLoS One. 2023 Mar 21;18(3):e0275734. doi: 10.1371/journal.pone.0275734 (PMC10030040; doi:10.1371/journal.pone.0275734)
Supplement: S16 Fig — Cluster I consists of agricultural pathogens. They were slightly 58 more abundant on the adaxial surface than the abaxial and were more prevalent on R. excelsa. (PDF) [file pone.0275734.s016.pdf]

S16 Fig

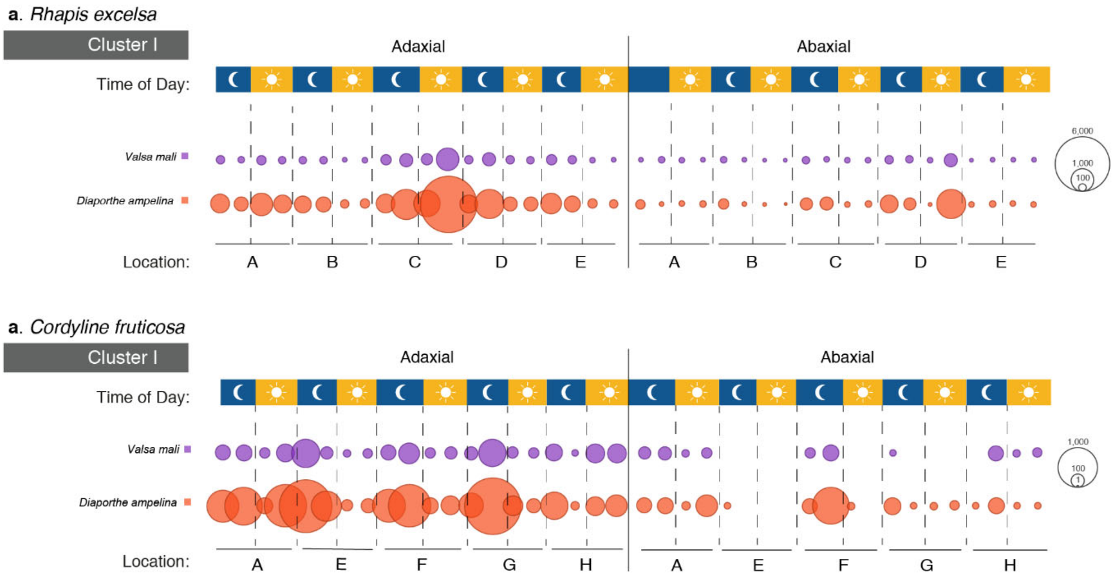

Microorganisms in Cluster I. Cluster I consists of agricultural pathogens. They were slightly more abundant on the adaxial surface than the abaxial and were more prevalent on *R. excelsa*.
