# Supplementary material for: Structure vs. chemistry: Alternate mechanisms for controlling leaf microbiomes
Source: PLoS One. 2023 Mar 21;18(3):e0275734. doi: 10.1371/journal.pone.0275734 (PMC10030040; doi:10.1371/journal.pone.0275734)
Supplement: S17 Fig — Cluster D comprises of agricultural pathogens and they were 62 slightly more abundant on the adaxial leaf surface. (PDF) [file pone.0275734.s017.pdf]

S17 Fig

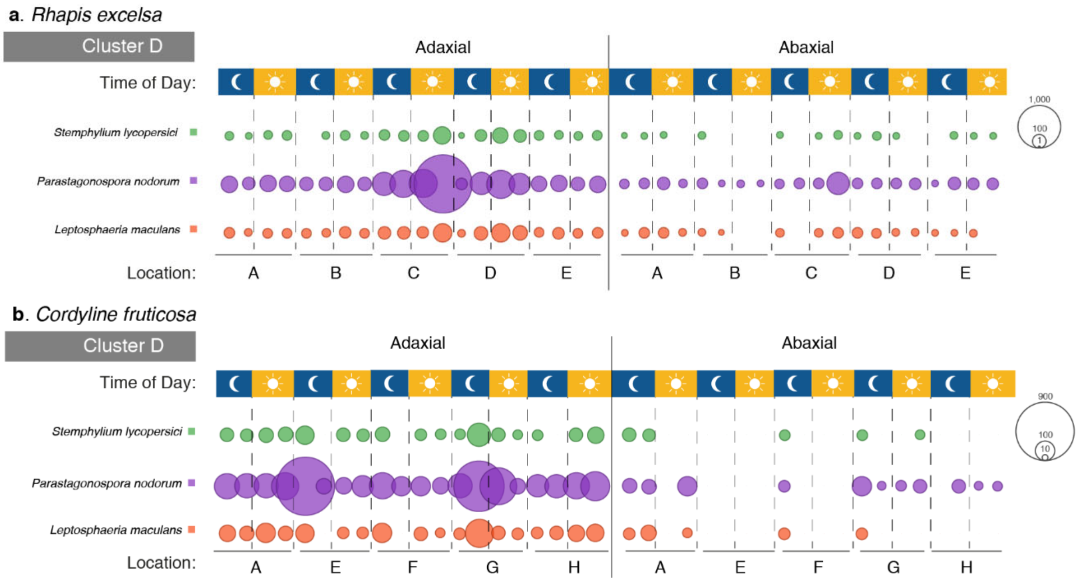

Microorganisms in Cluster D. Cluster D comprises of agricultural pathogens and they were slightly more abundant on the adaxial leaf surface.
