# Supplementary material for: Structure vs. chemistry: Alternate mechanisms for controlling leaf microbiomes
Source: PLoS One. 2023 Mar 21;18(3):e0275734. doi: 10.1371/journal.pone.0275734 (PMC10030040; doi:10.1371/journal.pone.0275734)
Supplement: S18 Fig — More than 2-fold increase 66 of ROS production on the abaxial surface of (a) R. excelsa. However, this phenomenon was 67 absent in (b) C. fruticosa. The fluorescence intensity readings of the ROS assay can be found 68 in S3 and S4 Tables. (PDF) [file pone.0275734.s018.pdf]

S18 Fig

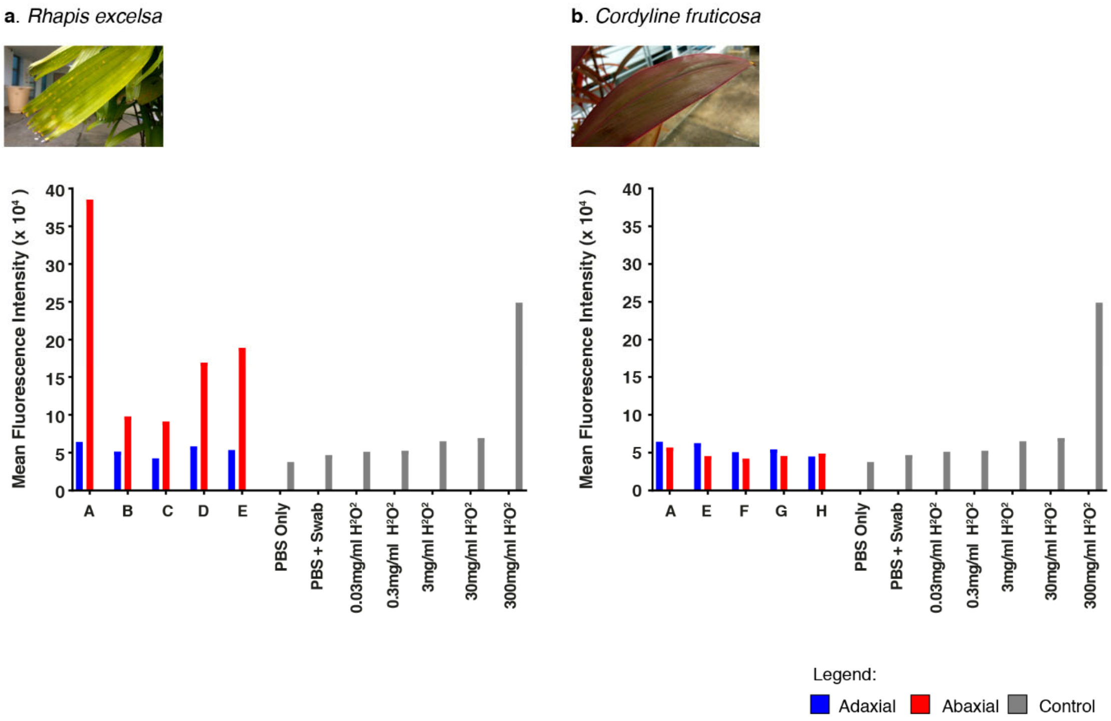

Reactive Oxygen Assay of Adaxial and Abaxial Leaf Surfaces. More than 2-fold increase of ROS production on the abaxial surface of (a) *R. excelsa*. However, this phenomenon was absent in (b) *C. fruticosa*. The fluorescence intensity readings of the ROS assay can be found in Table S3 and S4.
