# Supplementary material for: Structure vs. chemistry: Alternate mechanisms for controlling leaf microbiomes
Source: PLoS One. 2023 Mar 21;18(3):e0275734. doi: 10.1371/journal.pone.0275734 (PMC10030040; doi:10.1371/journal.pone.0275734)
Supplement: S1 Table — (PDF) [file pone.0275734.s019.pdf]

S1 Table. Pairwise relative distances in meters between sampling locations.

	A	B	C	D	E	F	G	H
A		968	875	838	564	826	607	331
B	968		942	1080	1380	1620	1380	720
C	875	942		190	830	956	787	980
D	838	1080	190		677	785	618	1010
E	564	1380	830	677		246	79	896
F	826	1620	956	785	246		248	1140
G	607	1380	787	618	79	248		914
H	331	720	980	1010	896	1140	914	

The global positioning system (GPS) coordinates of the sampling locations. Site A (1°21'02.7"N 103°40'49.0"E), B (1°21'15.2"N 103°41'18.7"E), C (1°20'45.1"N 103°41'15.0"E), D (1°20'41.0"N 103°41'10.0"E), E (1°20'43.8"N 103°40'46.0"E), F (1°20'36.3"N 103°40'44.7"E), G (1°20'42.5"N 103°40'49.3"E) and H (1°21'11.6"N 103°40'54.8"E).
