# Supplementary material for: Structure vs. chemistry: Alternate mechanisms for controlling leaf microbiomes
Source: PLoS One. 2023 Mar 21;18(3):e0275734. doi: 10.1371/journal.pone.0275734 (PMC10030040; doi:10.1371/journal.pone.0275734)
Supplement: S2 Table — (PDF) [file pone.0275734.s020.pdf]

S2 Table. Average number of reads generated.

	**Average No of Reads per Sample**															
**Location**	**A**		**B**		**C**		**D**		**E**		**F**		**G**		**H**	
**Reads**	**Raw**	**Trimmed**	**Raw**	**Trimmed**	**Raw**	**Trimmed**	**Raw**	**Trimmed**	**Raw**	**Trimmed**	**Raw**	**Trimmed**	**Raw**	**Trimmed**	**Raw**	**Trimmed**
***Rhapis excelsa* Top (Adaxial)**	3,274,633	3,261,104	2,845,651	2,835,429	3,107,821	3,089,430	3,447,060	3,433,598	3,328,104	3,303,744	NA	NA	NA	NA	NA	NA
***Rhapis excelsa* Bot (Abaxial)**	3,168,213	3,155,787	3,067,096	3,055,263	3,417,834	3,408,184	3,536,459	3,522,527	3,271,594	3,259,946	NA	NA	NA	NA	NA	NA
***Cordyline fruticosa* Top (Adaxial)**	3,469,239	3,453,876	NA	NA	NA	NA	NA	NA	3,491,641	3,452,866	3,268,662	3,255,603	3,371,341	3,354,795	3,260,118	3,249,589
***Cordyline fruticosa* Bot (Abaxial)**	3,097,181	3,084,474	NA	NA	NA	NA	NA	NA	3,299,476	3,286,264	2,917,482	2,874,268	3,465,699	3,445,998	3,148,736	3,135,073

Number of reads analysed for the adaxial and abaxial leaf surfaces at various locations. Raw reads were trimmed and filtered before performing further bioinformatic taxonomic analyses.
