# Supplementary material for: Structure vs. chemistry: Alternate mechanisms for controlling leaf microbiomes
Source: PLoS One. 2023 Mar 21;18(3):e0275734. doi: 10.1371/journal.pone.0275734 (PMC10030040; doi:10.1371/journal.pone.0275734)
Supplement: S3 Table — (PDF) [file pone.0275734.s021.pdf]

S3 Table. Mann Whitney test of *Chao-1* species richness estimates index between adaxial and abaxial leaf surface.

**Mann Whitney test**	**Bacteria**		**Fungi**	
Plant Species	*R. excelsa*	*C. fruticosa*	*R. excelsa*	*C. fruticosa*
p-value	<0.0001	<0.0001	0.0191	<0.0001
Median of Adaxial, n=20	93	147	82.5	75
Median of Abaxial, n=20	1	6	71.5	34.5
Difference: Actual	-92	-141	-11	-40.5
Difference: Hodges-Lehmann	-92	-137	-10	-34

There was a statistically significant reduction in an estimate of 92 and 137 bacterial species between the adaxial and abaxial surface on *R. excelsa* and *C. fruticosa* respectively. Reduction in fungal species between the two surfaces was also observed to be statistically significant but to a smaller extent at 10 and 34 species, respectively.
