# Supplementary material for: Structure vs. chemistry: Alternate mechanisms for controlling leaf microbiomes
Source: PLoS One. 2023 Mar 21;18(3):e0275734. doi: 10.1371/journal.pone.0275734 (PMC10030040; doi:10.1371/journal.pone.0275734)
Supplement: S4 Table — (PDF) [file pone.0275734.s022.pdf]

S4 Table. Raw fluorescence readings obtained from reactive oxygen species assay.

a. *Rhapis excelsa*

$\times 10^4$ notation	Locations				
Rhapis excelsa	**A**	**B**	**C**	**D**	**E**
Adaxial 1	6.65	4.83	4.23	4.37	4.88
Adaxial 2	6.21	5.48	4.28	7.25	5.81
Abaxial 1	60.2	71.5	10.8	18.1	12.6
Abaxial 2	16.9	12.4	74.4	15.7	25.3

b. *Cordyline fruticosa*

$\times 10^4$ notation	Locations				
Cordyline fruticosa	**A**	**E**	**F**	**G**	**H**
Adaxial 1	7.87	7.92	4.97	5.81	4.68
Adaxial 2	5.01	4.60	5.18	5.06	4.33
Abaxial 1	6.82	4.47	4.67	4.83	5.25
Abaxial 2	4.58	4.63	3.75	4.29	4.55

c. Controls

$\times 10^4$ notation

Negative Control	Replicate 1	Replicate 2
PBS Only	3.84	3.67
PBS + Sterile Swab	4.96	4.40
**H_2O_2 Standard Curve**		
0.03mg/ml	5.31	4.95
0.3mg/ml	5.35	5.14
3mg/ml	6.81	6.19
30mg/ml	7.01	6.86
300mg/ml	27.3	22.4

Fluorescence intensity readings of the adaxial and abaxial leaf surface of (a) *R. excelsa*, (b) *C. fruticosa* and (c) controls with duplicate samples taken from five different locations. The controls consist of duplicate negative controls of PBS and PBS with a sterile swab as well as positive controls with 10X serial dilution to make a standard curve of hydrogen peroxide.
