# Supplementary material for: Structure vs. chemistry: Alternate mechanisms for controlling leaf microbiomes
Source: PLoS One. 2023 Mar 21;18(3):e0275734. doi: 10.1371/journal.pone.0275734 (PMC10030040; doi:10.1371/journal.pone.0275734)
Supplement: S5 Table — (PDF) [file pone.0275734.s023.pdf]

S5 Table. Aggregate mean of adaxial, abaxial and control samples in the reactive oxygen species assay.

a. *Rhapis excelsa* (Expressed in $\times 10^4$ notation)

	Rhapis excelsa	A	B	C	D	E	PBS only	PBS+ Swab	0.03 mg/ml H_2O_2	0.3 mg/ml H_2O_2	3 mg/ml H_2O_2	30 mg/ml H_2O_2	300 mg/ml H_2O_2
Aggregate Mean	Adaxial	6.43	5.16	4.25	5.81	5.34	NA	NA	NA	NA	NA	NA	NA
	Abaxial	38.6	9.79	9.11	16.9	18.9	NA	NA	NA	NA	NA	NA	NA
	Control	NA	NA	NA	NA	NA	3.75	4.68	5.13	5.24	6.50	6.93	24.9

b. *Cordyline fruticosa* (Expressed in $\times 10^4$ notation)

	Cordyline fruticosa	A	E	F	G	H	PBS only	PBS+ Swab	0.03 mg/ml H_2O_2	0.3 mg/ml H_2O_2	3 mg/ml H_2O_2	30 mg/ml H_2O_2	300 mg/ml H_2O_2
Aggregate Mean	Adaxial	6.44	6.26	5.07	5.44	4.50	NA	NA	NA	NA	NA	NA	NA
	Abaxial	5.70	4.55	4.21	4.56	4.90	NA	NA	NA	NA	NA	NA	NA
	Control	NA	NA	NA	NA	NA	3.75	4.68	5.13	5.24	6.50	6.93	24.9

Aggregate mean fluorescence intensity readings of the adaxial and abaxial leaf surface of (a) *R. excelsa* and (b) *C. fruticosa*.
