# Supplementary material for: Structure vs. chemistry: Alternate mechanisms for controlling leaf microbiomes
Source: PLoS One. 2023 Mar 21;18(3):e0275734. doi: 10.1371/journal.pone.0275734 (PMC10030040; doi:10.1371/journal.pone.0275734)
Supplement: S6 Table — (PDF) [file pone.0275734.s024.pdf]

S6 Table. F-test and t-test of reactive oxygen species concentration between adaxial and abaxial leaf surface.

**F-test**	***Rhapis excelsa***	***Cordyline fruticosa***
Leaf surface	Adaxial VS Abaxial	Adaxial VS Abaxial
F-statistic	4.07E-09	0.175
p-value	$p < 0.05$	$p > 0.05$
**t-test**	**Welch's t-test (Unequal Variance)**	**Student's t-test (Equal Variance)**
t-statistic	0.0245	0.134
p-value	$p < 0.05$	$p > 0.05$
Statistically Significant	Significant	Not Significant

Statistically significant difference in ROS concentration was observed between the adaxial and abaxial surface in *Rhapis excelsa*.
