# Supplementary material for: Structure vs. chemistry: Alternate mechanisms for controlling leaf microbiomes
Source: PLoS One. 2023 Mar 21;18(3):e0275734. doi: 10.1371/journal.pone.0275734 (PMC10030040; doi:10.1371/journal.pone.0275734)
Supplement: S7 Table — (PDF) [file pone.0275734.s025.pdf]

S7 Table. Water contact angle measurements.

	Rhapis excelsa		***Cordyline fruticosa***	
	Left Contact Angle	Right Contact Angle	Left Contact Angle	Right Contact Angle
Adaxial	76.6°	70.9°	101.5°	98.7°
	85.5°	83.0°	110.5°	105.0°
	93.2°	92.4°	102.1°	103.2°
Average	83.6°		103.5°	
Abaxial	105.7°	98.3°	138.2°	142.3°
	106.0°	104.4°	138.8°	131.0°
	101.3°	94.9°	141.7°	138.6°
Average	101.8°		138.4°	

In addition to the image captured in Fig. 6, we took 3 additional contact angle readings. The adaxial surface of *R. excelsa* is likely to be hydrophilic, while its abaxial surface is slightly hydrophobic. The adaxial of *C. fruticosa* is slightly hydrophobic, while its abaxial surface is very hydrophobic due to its distinct hierarchical structure with micrometre scale folding and nanometre trichomes.
